# An “AND” Molecular Logic Gate as a Super‐Enhancers for De Novo Designing Activatable Probe and Its Application in Atherosclerosis Imaging

**DOI:** 10.1002/advs.202207066

**Published:** 2023-02-19

**Authors:** Mangmang Sang, Yibo Huang, Lu Wang, Lei Chen, Nawsherwan, Gang Li, Yan Wang, Xiu Yu, Cuilian Dai, Jinrong Zheng

**Affiliations:** ^1^ Institute of Cardiovascular Diseases Xiamen Cardiovascular Hospital of Xiamen University School of Medicine Xiamen University No. 2999 Jinshan Road, Huli District Xiamen 361006 China; ^2^ Nanjing Hospital of Chinese Medicine Affiliated to Nanjing University of Chinese Medicine Nanjing University of Chinese Medicine No. 157, Daming Road, Qinhuai District Nanjing 210000 China; ^3^ School of Pharmacy Gannan Medical University No. 1 Medical College Road, Zhanggong District Ganzhou 341000 China; ^4^ Shenzhen Key Laboratory of Respiratory Diseases Shenzhen People's Hospital Southern University of Science and Technology 3046 Shennan East Road, Luohu District Shenzhen 518055 China

**Keywords:** activatable probe, atherosclerosis, fluorescence imaging, molecular logic gates, peroxynitrite

## Abstract

Developing activatable fluorescent probes with superlative fluorescence enhancement factor (*F*/*F*_0_) to improve the signal‐to‐noise (S/N) ratio is still an urgent issue. “AND” molecular logic gates are emerging as a useful tool for enhanced probes selectivity and accuracy. Here, an “AND” logic gate is developed as super‐enhancers for designing activatable probes with huge *F*/*F*_0_ and S/N ratio. It utilizes lipid‐droplets (LDs) as controllable background input and sets the target analyte as variable input. The fluorescence is tremendously quenching due to double locking, thus an extreme *F*/*F*_0_ ratio of target analyte is obtained. Importantly, this probe can transfer to LDs after a response occurs. The target analyte can be directly visualized through the spatial location without a control group. Accordingly, a peroxynitrite (ONOO^−^) activatable probe (CNP2‐B) is de novo designed. The *F*/*F*_0_ of CNP2‐B achieves 2600 after reacting with ONOO^−^. Furthermore, CNP2‐B can transfer from mitochondria to lipid droplets after being activated. The higher selectivity and S/N ratio of CNP2‐B are obtained than commercial probe 3'‐(p‐hydroxyphenyl) fluorescein (HPFin vitro and in vivo. Therefore, the atherosclerotic plaques at mouse models are delineated clearly after administration with in situ CNP2‐B probe gel. Such input controllable “AND” logic gate is envisioned to execute more imaging tasks.

## Graphical abstract

An “AND” logic gate as super‐enhancers for designing activatable probes with huge *F*/*F*_0_ and S/N ratio. According to this logic gate, a peroxynitrite (ONOO^−^) activatable probe (CNP2‐B) is de novo designed. The *F*/*F*_0_ of CNP2‐B achieves 2600. This probe can accurately delineate the atherosclerotic plaques in vivo.

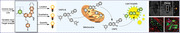

## 1 Introduction

Fluorescence imaging allow us to detect and uncover the physiological and pathological functions of an analyte of interest at the molecular level in a noninvasive, longitudinal manner.^[^ ^1^, ^2^ ^]^ The development of fluorescence probes has led to a better understanding and diagnosis of diseases such as cancer,^[^ ^3^ ^]^ parkinson's diseases,^[^ ^4^ ^]^ atherosclerosis,^[^ ^5^, ^6^, ^7^ ^]^ and bacterial infection.^[^ ^8^ ^]^ However, designing fluorescent probes with signal specificity and high signal‐to‐noise (S/N) ratio to deal with complex biological milieu is still an urgent issue.^[^ ^9^ ^]^ Although the efforts of extending the wavelength of the probe to the near‐infrared band can avoid the interference from biological background light,^[^ ^10^, ^11^ ^]^ it is still failed to eliminate the nonspecific fluorescence from the incompletely quenched probe. A novel fluorescence probe design principle needs to be developed to improve fluorescence quenching efficiency and enhance fluorescence enhancement factor (*F*/*F*_0_).

The probes that are activated to fluoresce only after interaction with a biomarker of interest give rise to specific interactions and enhanced signal‐to‐background ratio, improving the performance of fluorescence imaging for both in vitro and in vivo settings.^[^ ^12^, ^13^, ^14^ ^]^ A general strategy is to conjugate an organic fluorophore with a recognition moiety such that trapping or reacting with a specific biomarker or molecular event leads to the signal switching from an “off” state to an “on” state and, thus, indicates both the presence and the level of the biomarker.^[^ ^15^, ^16^, ^17^, ^18^ ^]^ However, each analyte need a special recognition group, which restrict the structural design of the probe. Therefore, it would be desirable to develop a simple and convenient principle able to improve the fluorescence quenching efficiency and assist in the accurate visual bioimaging. Inspired by the molecular logic‐based approaches raised by de Silva,^[^ ^19^ ^]^ we seek to develop an “AND” molecular logic gate that display significantly increased *F*/*F*_0_ and increased spatial resolution, which would help to enhance diagnostic and therapeutic accuracy.

**Scheme 1:**
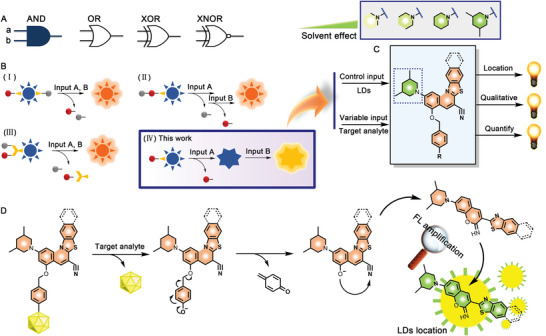
The “AND” molecular logic gate as super‐enhancers for de novo designing activatable probe. A) The type of molecular logic gate. B) The models of “AND” molecular logic gate. C) The structure of “AND” molecular logic gate for designing activatable probe. D) The activation mechanism of the “AND” molecular logic gate toward target analyte.

Molecular logic gates (MLGs) are compounds that can perform Boolean logic operations, such as AND, OR, XOR, and XNOR (**Scheme** **1**A), to give an answer (Output) upon receiving a stimulus (Input).^[^ ^20^, ^21^ ^]^ Among them, Molecular AND logic platform have been emerging as a useful tool for enhanced probe selectivity and signal‐to‐noise ratio. There are three established models (Scheme 1B): sequential activation,^[^ ^22^, ^23^ ^]^ parallel activation,^[^ ^24^, ^25^, ^26^ ^]^ and dual functionalization.^[^ ^27^, ^28^ ^]^ The dual functionalization strategy relies on two different triggering moieties anchoring two active sites in fluorescent reporter group. This obtained excellent fluorescence quenching efficiency, but limits the stability of the cargo backbone. To overcome this limitation, the activation of two triggers functionalized at a single active site has been developed: parallel activation and sequential activation. Although this mode can improve selectivity and accuracy with stable structure, it is no different from one‐to‐one molecular design principle in fluorescence quenching efficiency. Therefore, a generally applicable AND molecular logic gates with stable structure and high *F*/*F*_0_ still need to be developed.

In this study, we developed a simple and practical AND logic gate for designing activatable probes with high S/N ratio (Scheme 1C). This logic gate has only one active site. The recognition group of the probe was removed after responding with target analyte, and then the intramolecular charge rearrangement occurs to generate a coumarin derivative structure. This structure has a strong solvent effect. It can recognize lipid‐droplets (LDs) in cells, thus releasing fluorescence without secondary molecular adjustment (Scheme 1D). In this case, the fluorescence of the fluorophore is tremendously eliminated due to the quenching effect from double locking, thus an extreme *F*/*F*_0_ ratio is obtained by infinitely reducing *F*_0_. LDs as an organelle of lipid management are widely existed in various cells. We proposed a designing principle of AND logic probe with extremely signal‐to‐noise ratio that LDs can be used as controllable background input, and the target analyte acted as variable input. After a response occurs, this probe can transfer to LDs with enhanced *F*/*F*_0_ ratio. The analyte in complex biological microenvironment can be directly judged through the spatial location of fluorescence without a control group. This combination of spatial location and double locking strategy greatly improves the signal‐to‐noise ratio and effectively reduces the interference of background light from the probe.

## 2 Results and Discussion

### 2.1 Design and Spectroscopic Properties of the Probe

**Figure 1:**
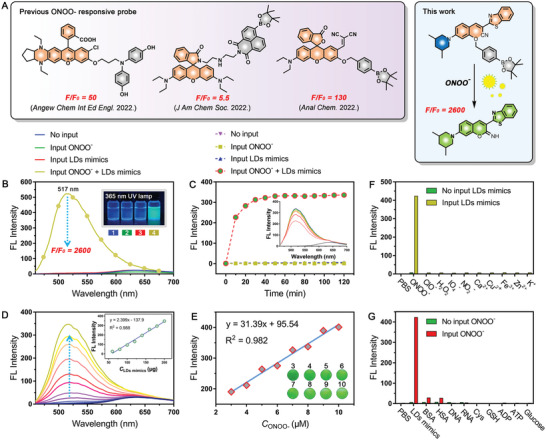
The responsiveness of CNP2‐B. A) The comparison of *F*/*F*_0_ between previous research and this work on ONOO^−^ responsive probe. B) The fluorescence spectrum of CNP2‐B (10 µm) in PBS (10 mm), ONOO^−^ (10 µm), LDs mimics (200 µg mL^−1^), and ONOO^−^ (10 µm) + LDs mimics (200 µg mL^−1^) solutions for 1 h. C) The fluorescence intensity of CNP2‐B (10 µm) at 517 nm under PBS (10 mm), ONOO^−^ (10 µm), LDs mimics (200 µg mL^−1^), and ONOO^−^ (10 µm) + LDs mimics (200 µg mL^−1^) solutions from 0 to 120 min. D) The fluorescence spectrum of CNP2‐B (10 µm) in different LDs mimics concentrations (0–200 µg mL^−1^) under 10 µm ONOO^−^for 1 h. E) The fluorescence intensity of CNP2‐B (10 µm) at 517 nm in different ONOO^−^ concentrations (3–10 µm) under 200 µg mL^−1^ LDs mimics. F) The fluorescence intensity of CNP2‐B (10 µm) at 517 nm in various biological species (PBS, 10 µm ONOO^−^, 100 µm ClO^−^, 100 µm H_2_O_2_, 100 µm IO_4_^−^, 100 µm NO_2_^−^, 100 µm Ca^2+^, 100 µm Cu^2+^, 100 µm Fe^3+^, 100 µm Zn^2+^, 100 µm K^+^) with 200 µg mL^−1^ LDs mimics. G) The fluorescence intensity of CNP2‐B (10 µm) at 517 nm in 200 µg mL^−1^ various reductive substances (PBS, LDs mimics, BSA, Human serum albumin (HSA), DNA, RNA, Cys, GSH, ADP, ATP, Glucose) with 10 µm ONOO^−^.

The AND type logic probe does not send signals when input A or input B exist alone, the fluorescent output is available only when the input A and input B exists at the same time. Accordingly, we use the LDs sequential controlled logic gate that performs AND function developed a peroxynitrite (ONOO^−^) responsive probe CNP2‐B. The synthetic route showed at Figures S1 and S2 in the Supporting Information. The fluorescence enhancement factor (*F*/*F*_0_) of CNP2‐B can reach 2600, which is much higher than the peroxynitrite probe developed in other researches^[^ ^29^, ^30^, ^31^ ^]^ (**Figure** **1**A).

For CNP2‐B probe, lipid droplet (LDs mimics) is the controllable input (background) and ONOO^−^ is the target analyte (variable input). As shown in Figure 1B, when LDs mimics or ONOO^−^ were input alone, the fluorescence signal of CNP2‐B was almost unchanged compared with the group without input. However, the fluorescence signal is significantly enhanced in the presence of LDs mimics and ONOO^−^. The *F*/*F*_0_ has reached an astonishing 2600 times at 517 nm. After irradiation with 365 nm UV lamp, the LDs mimics and ONOO^−^ double input groups shown strong green fluorescence, while the other three groups shown no fluorescence. The absorption spectrum has little change under different conditions (Figure S3, Supporting Information). Then the response speed of CNP2‐B was investigated (Figure 1C and Figure S4, Supporting Information); the response of CNP2‐B can reach two‐thirds within 10 min under the condition of double input of LDs mimics and ONOO^−^; the reaction is complete and all fluorescence signals are released within 30 min. The noninput group and single input group were investigated for 2 h, but the fluorescence of the three groups did not change. The above results proved that CNP2‐B shows excellent AND molecular logic performance.

To investigate the effect of LDs mimics and ONOO^−^ on the fluorescence emission of the CNP2‐B probe. First, the fluorescence intensity of CNP2‐B probe in different concentrations of LDs mimics was investigated under 10 µm ONOO^−^. The result shown in Figure 1D, it indicated that the fluorescence of CNP2‐B is concentration dependent on LDs mimics and shows a linear relationship (*y* = 2.399*x* − 137.9, *R* ^2^ = 0.988) within concentration range of 60–200 µg mL^−1^. Furthermore, the fluorescence intensity of CNP2‐B probe in different concentrations of ONOO^−^ was detected under 200 µg mL^−1^ LDs mimics. The response of CNP2‐B is also concentration dependent to ONOO^−^ (Figure 1E and Figure S5, Supporting Information), which linear correlation within the concentration range of 3–10 µm (*y* = 31.39*x* + 95.54, *R* ^2^ = 0.982). When the concentration of ONOO^−^ exceeds 10 µm, the fluorescence of CNP2‐B shows almost no change. The above results indicate that 10 µm CNP2‐B complete reaction with 10 µm ONOO^−^ by 1:1 reaction ratio.

The selectivity of CNP2‐B was investigated in many aspects. First, we investigate the potential interference of reactive oxygen species (ROS) and various ions on the responsiveness of two groups (CNP2‐B, CNP2‐B +LDs mimics). It was found that CNP2‐B only produced fluorescence response to ONOO^−^ and did not react with other biological species (Figure 1F and Figure S6A,B, Supporting Information), which demonstrated the probe with excellent selectivity to ONOO^−^. In the biological system, besides lipid, it also includes nucleic acid, protein, sugar, and other bioactive substances. To eliminate these disturbances, we investigate the potential interference of nucleic acid, protein, carbohydrate, etc. on the responsiveness of CNP2‐B. The result shown in Figure 1G and Figure S6C,D in the Supporting Information, it indicated that the fluorescence of CNP2‐B was slightly enhanced in the presence of BSA (bovine serum albumin) or human serum albumin (HSA), but there was still a huge gap compared with the LD mimics group. These results show that CNP2‐B has strong selectivity for ONOO^−^ and is suitable for responding to ONOO^−^ in complex physiological environment.

### 2.2 Response Mechanism of CNP2‐B

**Figure 2:**
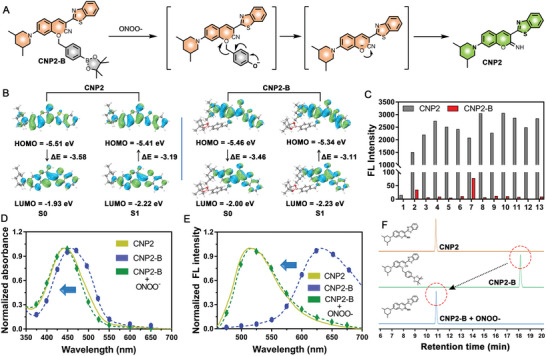
Response mechanism of CNP2‐B probe toward ONOO^−^. A) Mechanism diagram of CNP2‐B probe response to ONOO^−^. B) DFT‐calculation results of CNP2 and CNP2‐B probe. C) Fluorescence spectrum of CNP2 and CNP2‐B in different solvents (1–13 refer to PBS, DMF, DMSO, acetone, dichloromethane, dioxane, toluene, methanol, chloroform, ethanol, acetonitrile, ethyl acetate, and *N*‐butanol). D,E) Normalized absorption spectrum and fluorescence spectrum of CNP2, CNP2‐B, and CNP2‐B + ONOO^−^. F) Schematic diagram of high performance liquid chromatography (HPLC) in CNP2, CNP2‐B, and CNP2‐B + ONOO^−^.

The response mechanism of CNP2‐B was shown in **Figure** **2**A: ONOO^−^nucleophilic attack to borate ester site, causing boric acid to fall off and expose *p*‐phenol hydroxyl; After intramolecular electron rearrangement, the quenching group is completely detached from the fluorophore; Phenolic hydroxyl groups on exposed fluorophores attack cyano groups; Thus, the molecules form a ring to form a basic coumarin mechanism, which emits strong light in the lipid environment. To demonstrate this response mechanism, we adopted theoretical calculation and experimental verification.

First, the structure of CNP2‐B before and after response is calculated theoretically using time dependence‐density functional theory (DFT). The results are shown in Figure 2B, the highest molecular occupied orbital (HOMO) and lowest unoccupied orbital (LUMO) of CNP2 at S_0_ stage are −5.51 and −1.93 eV respectively with energy gap (Δ*E*) – 3.58 eV; HOMO and LUMO in S_1_ stage are −5.46 and −2.00 eV respectively with energy gap (Δ*E*) −3.19 eV. The HOMO and LUMO of CNP2‐B at S_0_ stage are −5.51 and −1.93 eV respectively with energy gap (Δ*E*) – 3.58 eV; However, HOMO and LUMO in S_1_ stage are −5.34 and −2.23 eV respectively with energy gap (Δ*E*) −3.11 eV. As can be seen from the above data, after CNP2‐B responds to ONOO^−^ and changes to CNP2, HOMO‐LUMO bandgap increases, the increase of the energy required for the transition results in the blueshift of the absorption wavelength. The HOMO and LUMO orbital energy levels of CNP2 are higher than those of CNP2‐B, illustrated that the typical probe molecules are more obvious as electron donors, and are more susceptible to solvent effects due to the influence of polar solvents.

Then, the solvent effects of CNP2 compared with CNP2‐B shown at Figure 2C and Figures S7 and S8 in the Supporting Information. The results showed that the fluorescence of CNP2 was stronger than that of CNP2‐B in different solvents and the solvent effect was more obvious. Normalize the absorption spectra and emission spectra of CNP2 and CNP2‐B before and after response (Figure 2D,E), the absorption peak and emission peak of CNP2‐B are redshifted than those of CNP2, and the emission peak redshifts by nearly 140 nm. The absorption spectrum and emission spectrum of CNP2‐B after response are basically consistent with those of CNP2, proved that the product of CNP2‐B after response is CNP2.

The results of high resolution mass spectrometry(HRMS) also prove that the product of CNP2‐B after response is CNP2 (Figure S9, Supporting Information). Figure 2F and Figure S10 in the Supporting Information showed the CNP2 mass spectrum peak detected in the CNP2‐B response product and the reaction is almost complete with very little CNP2‐B residues. By analyzing the retention time of the CNP2‐B response product proved that CNP2‐B response with ONOO^−^ did generate CNP2 after responding. The above data illustrated that CNP2‐B reacts with ONOO^−^ and generates CNP2 through intramolecular electron rearrangement and cyclization.

### 2.3 The Responsiveness and Organelle Localization of CNP2‐B in Cells

**Figure 3:**
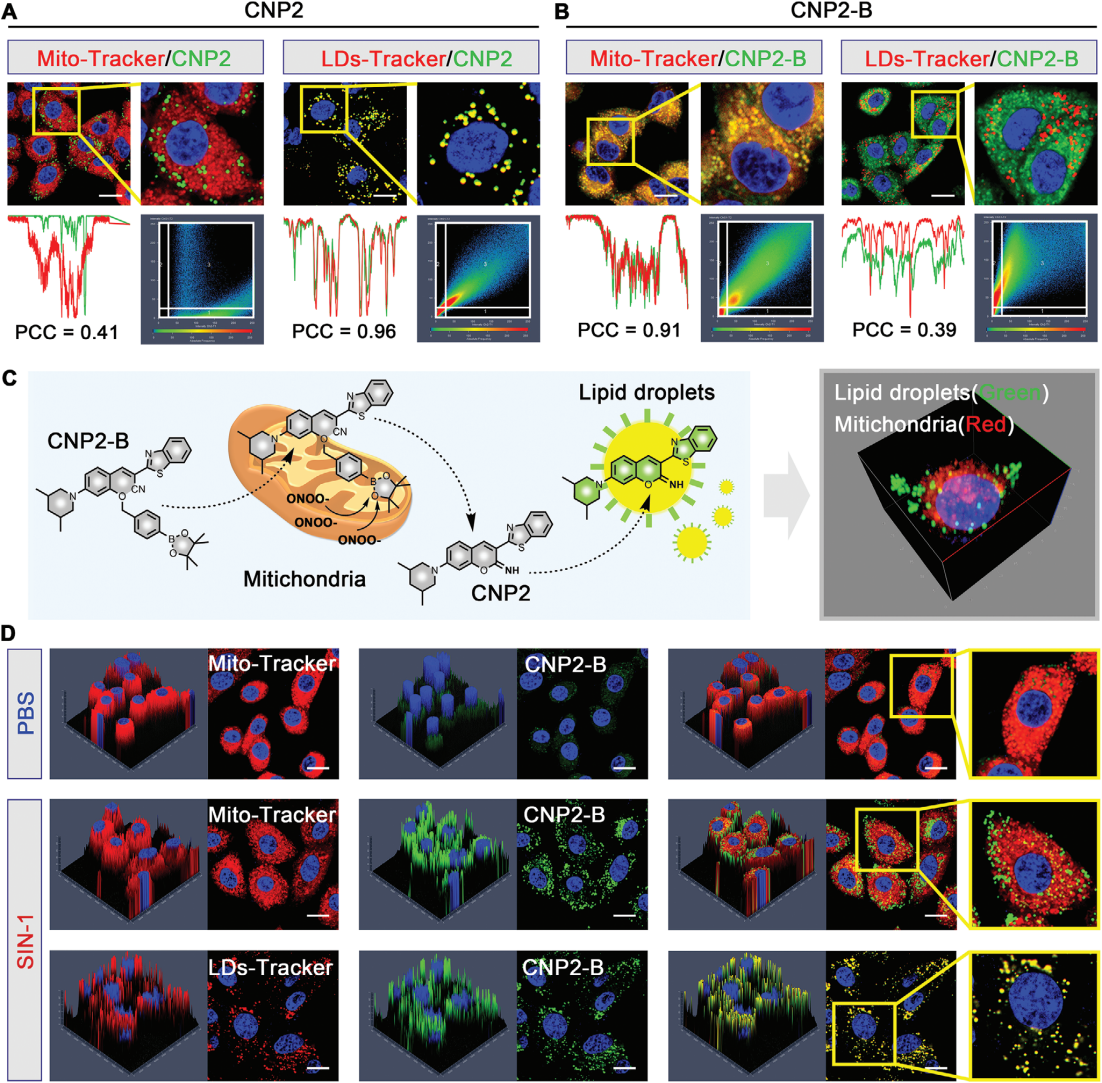
CNP2‐B transforms into CNP2 after responding to ONOO^−^ in mitochondria, and then transfers to LDs with strong fluorescence. A) The confocal imaging of A549 cells were obtained after the coincubation of Mito‐Tracker or LDs‐Tracker with CNP2 (10 µm) for 1 h. B) The confocal imaging of A549 cells were obtained after the coincubation of Mito‐Tracker or LDs‐Tracker with CNP2‐B (10 µm) for 1 h. C) The structural and organelle localization changes of CNP2‐B after entering the cell. D) The A549 cells were pretreated with 20 µm SIN‐1 for 1 h and then incubated with CNP2‐B (10 µm), Mito‐Tracker or LDs‐Tracker for 1 h. Green channel: *λ*_ex_ = 488 nm, red channel: *λ*_ex_ = 555 nm, and blue channel: *λ*_ex_ = 405 nm. Scale bars: 10 µm.

The principle of lipid droplet imaging of the CNP2‐B probe in cells as shown in **Figure** **3**A, that the probe enters the mitochondria and then responds to ONOO^−^, and changing to CNP2, with lipid droplet imaging function. In order to evaluate the cellular imaging ability and organelle localization of CNP2 and CNP2‐B, we selected A549 cells with clear lipid droplets as the tool cell. The A549 cells were incubated with CNP2 and commercial lysosomal probe (Lyso‐Tracker), mitochondrial probe (Mito‐Tracker), endoplasmic reticulum probe (ER‐Tracker), and lipid drop probe (LDs‐Tracker) for 1 h, respectively. Confocal microscope is used to analyze the colocation of probe and organelle.

The results show that CNP2 anchored to lipid droplets, and the colocalization coefficient with the lipid droplets probe is up to 0.96. However, the colocalization coefficients of CNP2 with lysosomal probe, mitochondrial probe, and endoplasmic reticulum probe were 0.32, 0.41, and 0.54, respectively (Figure 3A and Figures S11–S13, Supporting Information). That indicates the CNP2 probe is mainly located in lipid droplets in cells. The results as Figure 3B and Figures S14 and S15 in the Supporting Information indicate that the colocalization coefficient of CNP2‐B with mitochondria was 0.91, and that of CNP2‐B with lipid probe only 0.39, which indicated that CNP2‐B has mitochondrial localization function before response with ONOO^−^. According to these results, we speculate that the unresponsive state of CNP2‐B is mitochondrial localization, and after the response with ONOO^−^, the structure changes to CNP2 and then transfers to lipid droplets (Figure 3C). To test this conjecture, we use the common ONOO^−^ donor reagent 3‐Morpholinosydnonimine (SIN‐1)20 µm to prostimulate A549 cells for 2 h to make enough ONOO^−^ in cells, then coincubated with CNP2‐B and other probes for another 1 h. It can be seen from Figure 3D that CNP2‐B lost its mitochondrial localization function after SIN‐1 was pre‐incubated, and the fluorescence was transferred to lipid droplets with significantly enhanced.

In addition, the mitochondrial health index is usually reflected by enzyme activity of citrate synthase, cytochrome Coxidase (CCO), and succinate dehydrogenase (SD). Therefore, we use the enzyme activity assay kit to evaluate the potential mitochondrial toxicity of CNP2‐B. The results showed that CNP2‐B has no mitochondrial toxicity at the concentration of 10 µm, but the enzyme activity showing a certain decreasing when the concentration achieves 100 µm (Figure S16, Supporting Information). These results indicated that the concentration of 10 µm CNP2‐B is a safe dosage for mitochondrion.

The above results lead to an interesting conclusion, in addition to the change of fluorescence intensity, CNP2‐B also has the function of organelle transfer before and after response with ONOO^−^. The classical fluorescence probe generally judges the concentration of the substance to be measured by comparing the fluorescence intensity of the experimental group with that of the control group. However, the fluorescence intensity is easily interfered by many factors, such as drug concentration, cell status, background light, etc. The method of combining spatial positioning with quantitative analysis is more reliable than that of relying solely on the change of fluorescence intensity to determine the degree of response. It can not only judge the response degree of the probe by comparing the fluorescence intensity with the blank control group but also directly determine whether the response occurs by directly locating the fluorescence space on a single cell. Therefore, if the fluorescence of CNP2‐B is located in lipid droplets after administration, we can determine that there is enough ONOO^−^ in the cells. In addition, the concentration of ONOO^−^ in cells can be semi quantified by comparing the fluorescence intensity in lipid droplets with the control group. This brings a new strategy for detecting the concentration of ONOO^−^ in cells.

### 2.4 CNP2‐B Specific Recognition of Foam Cells

**Figure 4:**
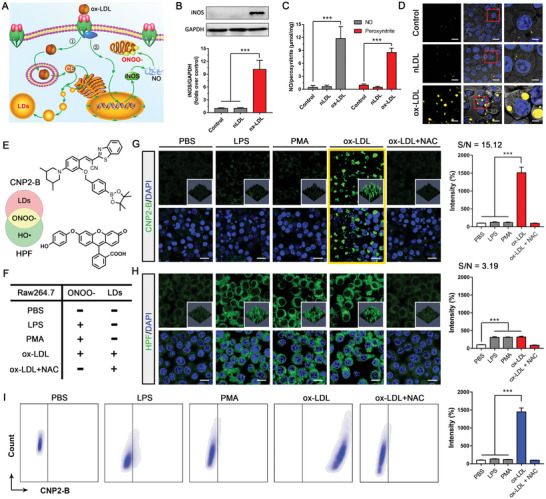
CNP2‐B probe specific recognition of macrophage derived foam cells. A) The mechanism of ox‐LDL induces macrophages transform to foam cells with the abnormal accumulation of LDs and peroxynitrite. B) The expression of iNOS in RAW 264.7 cells after stimulating by 10 µg mL^−1^ nLD or ox‐LDL (*n* = 6; mean ± SD, ***P < 0.001). C) NO and ONOO^−^ content in RAW 264.7 cells after stimulating by 10 µg mL^−1^ nLDL or ox‐LDL (*n* = 6; mean ± SD, ***P < 0.001). D) Fluorescence imaging of LDs‐Trackerin RAW 264.7 cells after stimulating by 10 µg mL^−1^ nLDL and ox‐LDL. E) The structure and target analyte of CNP2‐B and HPF. F) The content of ONOO^−^and LDs in RAW 264.7 cells after stimulating with LPS, PMA, ox‐LDL, or ox‐LDL + NAC. G) Confocal fluorescence imaging of RAW 264.7 cells stained by 10 µm CNP2‐B under different stimulants (PBS, LPS, PMA, ox‐LDL, ox‐LDL + NAC) (*n* = 6; mean ± SD, ***P < 0.001). H) Confocal fluorescence imaging of RAW 264.7 cells stained by 10 µm HPF under different stimulants (PBS, LPS, PMA, ox‐LDL, ox‐LDL + NAC) (*n* = 6; mean ± SD, ***P < 0.001). I) Flow analysis of RAW 264.7 cells incubated with CNP2‐B under different stimulants (PBS, LPS, PMA, ox‐LDL, ox‐LDL + NAC) (*n* = 6; mean ± SD, ***P < 0.001).

Macrophages are the core component of atherosclerotic plaque, which are induced by local inflammation of blood vessels to recruit into the subendothelial layer and form foam cells under the stimulation of oxidized low density lipoprotein (ox‐LDL). As shown in **Figure** **4**A: ox‐LDL acting on macrophages can activate a variety of cellular activities, including the decomposition and release of free cholesterol in lysosomes, and forming lipid droplets through the endoplasmic reticulum. And ox‐LDL can induce macrophages to express iNOS, thereby promoting the production of NO, which reacts with superoxide anion to generate ONOO^−^. In this process, foam cells generated two input conditions of CNP2‐B, lipid droplet, and ONOO^−^. Therefore, we can use CNP2‐B to carry out specific fluorescence imaging on foam from macrophage and pathological activity of atherosclerotic plaque.

First, the model of ox‐LDL inducing macrophages to foam cells was evaluated. Using ox‐LDL (10 µg mL^−1^) stimulate RAW 264.7 cells for 24 h extracted protein from cells for western blot analysis. The results (Figure 4B) have shown that the iNOS protein expression in cells was significantly increased after ox‐LDL stimulation. And the total ROS, NO, and ONOO^−^content in cells increased significantly (Figure 4C and Figure S17, Supporting Information). The lipid droplets in macrophages are very small under physiological conditions, while the lipid droplet volume in cells increases rapidly (Figure 4D) after ox‐LDL stimulation. These results proved that using ox‐LDL to stimulate macrophages can form foam cells, accompanied by abnormal accumulation of ONOO^−^ and lipid droplets, which created the basic conditions for using CNP2‐B to detect foam cells and plaque formation.

In order to demonstrate the excellent imaging ability of CNP2‐B in cellular, we use commercial ONOO^−^ probe hydroxyphenyl fluorescein (HPF) to compare with CNP2‐B in foam cell imaging capabilities (Figure 4E). The macrophages were stimulated with different stimulators to make them have different content of ONOO^−^ and lipid droplet (Figure 4F): phosphate buffer solution (PBS) group was the control group without ONOO^−^ and lipid droplet; Lipopolysaccharide (LPS) stimulation group can induce the increase of ONOO^−^ content, but does not form lipid droplet; Phorbol 12‐myristate 13‐acetate (PMA) can also induce the increase of ONOO^−^ content without forming lipid droplets; ox‐LDL stimulation can stimulate cells to produce ONOO^−^ and lipid droplets simultaneously; after treatment with antioxidant NAC (N‐acetyl‐l‐cysteine), ROS in cells was cleared, and there was no effect on lipid droplets induced. Figure 4G–I indicates that the fluorescence was enhanced in CNP2‐B group stimulated cells with obvious spatial location only under the stimulation of ox‐LDL compared with other four groups and its S/N ratio up to 15.12. However, HPF can enhance fluorescence in cells under LPS (lipopolysaccharide), PMA (phorbol 12‐myristate 13‐acetate), and ox‐LDL stimulation, and its S/N ratio is only 3.19. These results illustrated that CNP2‐B can accurately recognize ox‐LDL induced foam cell formation under the stimulation of multiple inflammatory signals. The logic probe using lipid droplets as control input has great advantages in improving the S/N ratio and reducing background interference, which provides an application basis for applying of CNP2‐B to plaque imaging in vivo.

### 2.5 Imaging Performance of CNP2‐B at Tissue Level and the Preparation of In Situ Probe Gel

**Figure 5:**
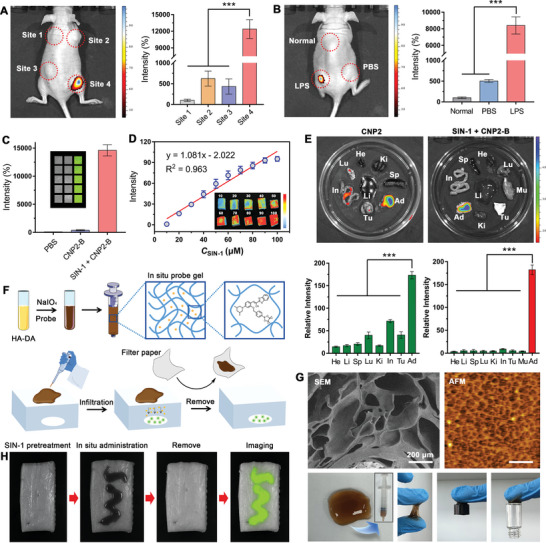
Imaging performance of CNP2‐B at tissue level and the preparation of in situ probe gel. A) Fluorescence imaging of BALB/c nude mice after inject with different reagents on the back of mice (site 1: CNP2‐B dissolved in PBS solution, site 2: CNP2‐B dissolved in sunflower seed oil solution, site 3: pro‐injected with SIN‐1 and then injected with PBS solution of CNP2‐B, site 4: pro‐injected with SIN‐1 and then injected with sunflower seed solution of CNP2‐B) (*n* = 6; mean ± SD, ***P < 0.001). B) Fluorescence imaging of BALB/c nude mice after injection with PBS and LPS on the back of mice, and then injected with sunflower seed solution of CNP2‐B (*n* = 6; mean ± SD, ***P < 0.001). C) Fluorescence imaging of adipose tissue after incubated with CNP2‐B and CNP2‐B+ SIN‐1 (*n* = 6; mean ± SD). D) Fluorescence intensity of adipose tissue after incubated with SIN‐1 of different concentrations (10–100 µm) (*n* = 6; mean ± SD). E) Fluorescence imaging of different tissues (heart, liver, spleen, lung, kidney, intestine, tumor, and adipose) from mice after treating with 100 µm SIN‐1 and CNP2‐B (*n* = 6; mean ± SD, ***P < 0.001). F) Synthesis and administration of in situ probe gel. G) Transmission electron microscope (TEM), atomic force microscope (AFM), and photographs of hydrogels. H) Fluorescence imaging of in situ CNP2‐B gel coated adipose tissue.

BALB/c nude mice were used to investigate the imaging performance of CNP2‐B probe in vivo. Set 4 sites on the back of the mouse: CNP2‐B dissolved in PBS and injected into site 1; CNP2‐B dissolved in sunflower seed oil and inject into site 2; proinjected with SIN‐1 solution into site 3, and then inject PBS solution of CNP2‐B; proinjected with SIN‐1 solution into site 4, and then inject sunflower seed solution of CNP2‐B. As shown in **Figure** **5**A, site 1, 2, and 3 hardly emit fluorescence using living imaging system, however, site 4 can emit strong fluorescence, which is about 120 times stronger than other three sites. LPS solution was injected subcutaneously to induce endogenous ONOO^−^ production. Compared with the site injected with PBS, fluorescence from LPS injected site was significantly enhanced to 80 times (Figure 5B). The above results illustrated that both endogenous and exogenous ONOO^−^ can be detected by CNP2‐B probe with lipid assistance in vivo.

The fresh adipose tissues of pig were used  to evaluate the responsiveness of CNP2‐B to lipid and ONOO^−^. Divide the fat tissue into three groups: the first group was immersed in PBS; the second group was immersed in PBS solution of CNP2‐B; the third group was incubated with CNP2‐B after soaking in SIN‐1 solution for 1 h. Using living imaging system to analyze the fluorescence intensity of adipose tissues. The results are shown in Figure 5C, CNP2‐B can emit strong fluorescence only under the condition of the adipose tissue is pretreated with SIN‐1, indicate that CNP2‐B will not respond if it lacks ONOO^−^in vivo. Then we studied the reaction kinetics of CNP2‐B at the adipose tissue. Fresh adipose tissue was prepared with the same size, immersed in different concentration of SIN‐1 solution for 1 h, and then coincubated with the 10 µm CNP2‐B probe. Fluorescence imaging analysis was performed in tissues and statistical fluorescence signals. The results are shown in Figure 5D, there is a linear correlation (*y* = 1.081*x* − 2.022, *R* ^2^ = 0.963) between fluorescence intensity and SIN‐1 with concentration range of 10–100 µm. We investigated fluorescence imaging of different tissues incubated with CNP2‐B probe. The results are shown in Figure 5E, after ONOO^−^ donor reagent SIN‐1 pretreatment, the organ tissues was coincubated with CNP2‐B probe, and the fluorescence imaging contrast between adipose tissue and other tissues was more obvious. These results demonstrated that the CNP2‐B probe still has ONOO^−^ response ability in tissues, provide basis for fluorescence imaging in vivo.

In order to facilitate the in situ fluorescence imaging of probe at the site to be measured in the living body, a kind of in situ probe hydrogel based on HA‐DA (hyaluronic acid graft dopamine) material was developed. With the aid of ultrasound, HA‐DA was dissolved in PBS, crosslinking agent NaIO_4_ and PBS solution of CNP2‐B probe were added, then the stable probe‐loaded hydrogel was obtained after 2 h (Figure 5F). The structure of hydrogel was characterized by scanning electron microscope (SEM) and atomic force microscope (AFM). Figure 5G and Figure S18 in the Supporting Information showed that the hydrogel with a porous structure is conducive to the storage of CNP2‐B probe solution and penetration into target tissues. The hydrogel is dark brown with smooth surface and can be sucked and contained in the syringe, it has self‐healing and injectable property. A large amount of crosslinked dopamine in HA‐DA has a polyphenolic structure, with strong wet adhesion ability, which helps the hydrogel tightly bind to the target tissue and release the encapsulated probe.

The efficient of hydrogel release probe CNP2‐B was measured. We place the CNP2‐B loaded hydrogel in PBS and test the absorption intensity of the probe in PBS solution at regular intervals. As Figure S19 in the Supporting Information showed, the probe is gradually released from the hydrogel into the solution with the extension of the time. The release rate of probe within 10 min is 35.9%. The plaque is located in the middle of the arterial wall. Therefore, the probe only needs to pass through a layer of vascular smooth muscle cells (VSMCs) to reach the plaque when in situ administration. We simulated such a process in vitro through transwell assay (Figure S20A, Supporting Information). To simulate plaque, the SIN‐1 coincubated mouse adipose tissue was place in the lower chamber, and then culture of a layer of human aortic smooth muscle cells in the upper chamber. The adipose tissue was light up after administration with CNP2‐B loaded hydrogel and SIN‐1 (Figure S20B, Supporting Information). This indicated that the CNP2‐B can pass through VSMCs and transfer from hydrogel to plaque.

To investigate the probe release ability of hydrogel in vitro, a fresh adipose tissue was prepared. After pretreatment of adipose tissue with SIN‐1, a syringe was used to coat a specified shape on the surface of tissue with CNP2‐B probe‐loaded hydrogel. After standing for 10 min, filter paper was used to absorb and remove the probe‐loaded hydrogel. Then the fat tissue was put into living imaging system to observe the fluorescence. The results are shown in Figure 5H, the tissue coated with probe‐loaded hydrogel emit strong fluorescence, proved that the probe infiltrated into the tissue from the hydrogel and generates fluorescence after response with lipid and ONOO^−^. These results illustrated that the developed probe‐loaded hydrogel was suitable for the CNP2‐B probe administered to local tissues, provide evidence for vascular tissue administration with probe in vivo.

### 2.6 In Vivo Atherosclerotic Plaque Imaging

**Figure 6:**
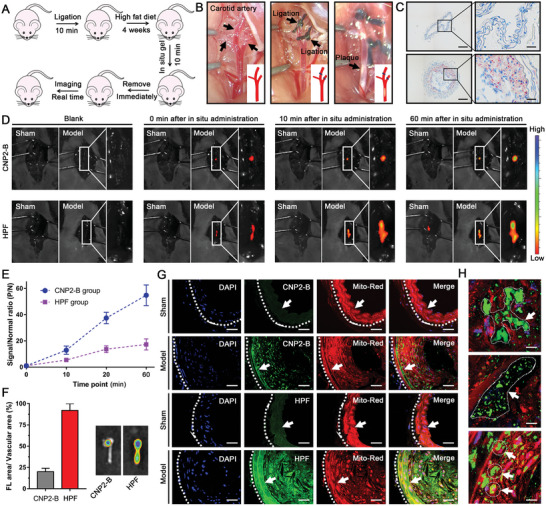
In vivo imaging of atherosclerotic plaque by CNP2‐B. A) Flow chart of fluorescence imaging of atherosclerosis in mice. B) Construction of surgical model of carotid atherosclerosis. C) Oil red O stain images of carotid artery blood vessel. Scale bars, 100 µm. D, E) Fluorescence imaging of carotid atherosclerosis with in situ gel contain HPF or CNP2‐B. *λ*_ex_ = 460 nm (*n* = 6; mean ± SD). F) Ex vivo imaging of carotid atherosclerosis blood vessel administration by HPF or CNP2‐B (*n* = 6; mean ± SD). G,H) Confocal imaging of freeze‐sectioned left carotid artery. Green channel: *λ*_ex_ = 488 nm, red channel: *λ*_ex_ = 555 nm, and blue channel: *λ*_ex_ = 405 nm. Scale bar = 100 µm.

To investigate the fluorescence imaging of atherosclerotic plaque by CNP2‐B probe in vivo, using ApoE mice make a carotid artery atherosclerosis plaque model by carotid artery ligation combined with high‐fat diet. The operation process of using carotid artery atherosclerosis plaque model to evaluate the fluorescence imaging performance of probe‐loaded hydrogel for atherosclerotic plaque is summarized in **Figure** **6**A. There are three branches at the upper edge of the right carotid arteries, keep the thinner branches and ligate the two thicker branches to limit blood flow (Figure 6B). This angiostenosis procedure increases the blood pressure and blood flow speed of the lower carotid artery, combined with high‐fat diet, the pathological environment of plaque formation can be simulated. The right carotid artery slices stained with oil red O as Figure 6C, the accumulation of lipid on the vascular wall, plaque formation and stenosis of the vascular lumen were clearly distinguish. The above results indicate that the carotid atherosclerosis model was successfully constructed.

We performed the in vivo imaging of carotid atherosclerosis model after injecting 200 µL CNP2‐B (10 µm) via tail vein. But it can hardly detect effective signals at the carotid artery (Figure S21, Supporting Information). To investigate the fluorescence imaging ability of CNP2‐B probe‐loaded hydrogel for atherosclerotic plaque, carotid atherosclerosis model was constructed. The probe‐loaded hydrogel was applied on the surface of the carotid artery blood vessel in situ, after 10 min of standing, hydrogel was removed with filter paper, and continuous observed by living imaging system within 1 h. As shown in Figure 6D,E, fluorescent signals can be observed at the carotid artery after removing the probe‐loaded hydrogel. Then the fluorescence gradually increased, and the CNP2‐B group could clearly indicate the plaque position on the blood vessel after 10 min. There was no significant difference in fluorescence imaging time between CNP2‐B group and HPF (an inflammation response fluorescent probe) group. The carotid arteries in the sham operation group also showed a certain intensity of fluorescence after the application of HPF, while the S/N ratio of HPF group is lower. The nonspecific fluorescence of CNP2‐B group is lower, which greatly improves the S/N ratio to 55 after 1 h of application. Then remove the carotid artery and observe the fluorescence distribution (Figure 6F): the fluorescence was relatively concentrated in CNP2‐B group, and only the lump fluorescence was detected in the local blood vessels, which was similar to the distribution of vascular plaque. However, fluorescence was distributed in almost the whole blood vessel in HPF group, indicated that HPF probe has poor selectivity to plaque.

The probe stained carotid artery was frozen and observed with confocal microscope. As shown in Figure 6G, the fluorescent signal of CNP2‐B could hardly be detected in the vascular wall of the sham operation group, while the green fluorescent clumps can be observed at the plaque in the model group, which indicated a large amount of lipid droplets and lipid clumps in the plaque. Although the differential fluorescence signal of HPF probe can also be detected in the sham operation group and the model group, the fluorescence signal is scattered and cannot distinguish specific cell tissues. Enlarge the frozen sections of CNP2‐B group, the accumulation of lipid in the lower endothelium and the core of lipid necrosis are clearly visible (Figure 6H). These results illustrated that CNP2‐B probe is specific for plaque in vivo, the probe design based on “AND” molecular‐scale logic gates functional lipid controllable input has outstanding potential in improving signal‐to‐noise ratio and reducing background interference.

### 2.7 Biosafety of CNP2‐B

The biosafety was carefully evaluated. First, the cell viability of A549 and Raw 264.7 were tested by thiazolyl blue (MTT) after administration with CNP2‐B, HA‐DA hydrogel, or CNP2‐B loaded hydrogel. The CNP2‐B is no toxicity within 20 µm, when the concentration reaches 100 µm, the cell viability decreases by about 30% (Figure S22A, Supporting Information). The HA‐DA hydrogel has high biological safety (Figure S22B, Supporting Information). However, it has slight toxicity at high concentration after loading the probe CNP2‐B (Figure S22C, Supporting Information). Secondarily, the hepatotoxicity, nephrotoxicity, and routine blood tests showed that 10 µm CNP2‐B or CNP2‐B loaded hydrogel has no effect on blood, liver, and kidney functions (Figures S23 and S24, Supporting Information). Moreover, the histomorphology of important organs is normal (Figure S25, Supporting Information). In addition, we investigated the effect of CNP2‐B on hemolysis, the results showed that CNP2‐B will not cause hemolysis within 20 µm (Figure S26, Supporting Information). Finally, the anatomical morphology of carotid artery was inspected, the results showed that carotid artery is normal after in situ administration with CNP2‐B loaded hydrogel (Figure S27, Supporting Information). The above data indicated that the HA‐DA hydrogel has high safety. Although the probe has certain toxicity at high concentration, it has no toxicity at 10 µm administration concentration. Therefore, the probe is suitable for in vivo and in vitro imaging.

To examine the potential damage of carotid artery after in vivo imaging, the carotid artery was exposed and repeated the in vivo imaging process for 0, 10, and 100 cycles, respectively. The H‐E staining and Masson stain was conducted, the results were showed at Figure S28 in the Supporting Information. The anatomical morphology and collagen deposition of vascular wall was normal after in vivo imaging within 100 cycles. But the total ROS content of carotid artery was slightly increasing after 100 consecutive testing (Figure S29, Supporting Information). The slight change of ROS can be ignored during image‐guided surgery, because the surgery can cause huge traumatic inflammation, so anti‐inflammatory treatment will be carried out. However, we must avoid direct expose the eyes to blue light during the operation.

To evaluate the long‐term biosafety of the probe CNP2‐B, the pharmacokinetic behavior and organs toxicity was studied. First, the pharmacokinetic behavior of CNP2‐B after intravenous injection (5 mg kg^−1^) accorded with the single compartment model. The concentration–time curve was shown at Figure S30 in the Supporting Information, the probe was almost cleared within 24 h. The pharmacokinetic parameters of the probe were calculated in Table S1 in the Supporting Information: the elimination half‐time (*t*_1/2_) of the probe was 0.22 h; the apparent distribution volume (*V*) of the probe was 1.09 mg µg^−1^ mL^−1^; the clearance of probe was 3.38 mg µg^−1^ mL^−1^)/h; the area under the curve (AUC_0–_ *_t_*) of the probe was 1.47 µg mL^−1^ h^−1^; the mean retention time of the probe was 0.32 h. Second, the anatomical morphology of organs was normal after caudal vein injection of CNP2‐B (100 µL, 10 µm) for 5 d (Figure S31, Supporting Information); furthermore, the value of alanine aminotransferase/ glutamic transaminase (ALT/AST,hepatotoxicity) and creatinine/ urea (CREA/UREA, nephrotoxicity) was normal (Figure S32, Supporting Information). These results indicated that the probe CNP2‐B can be cleared quickly and is safety after administration.

## 3 Conclusion

In summary, we have developed a simple and practical “AND” logic gate as super‐enhancers for designing activatable probes with huge *F*/*F*_0_ and S/N ratio. This “AND” logic gate utilized LDs as controllable background input, and set the target analyte as variable input. According to this principle, we de novo designed a peroxynitrite (ONOO^−^) activatable probe (CNP2‐B). The *F*/*F*_0_ of CNP2‐B achieved 2600 after reacting with ONOO^−^. Interestingly, CNP2‐B can transfer from mitochondria to lipid droplets after being activated. The higher selectivity and S/N ratio of CNP2‐B were obtained than commercial probe HPF in vitro and in vivo. Based on the large presence of lipid and ONOO^−^ in atherosclerotic plaque, a probe‐loaded hydrogel was constructed for better plaque imaging. This probe has a highly sensitive fluorescence imaging ability in cellular and mouse models. It is worth noting that this probe can detect the ONOO^−^ content accurately in complex biological samples, which is conducive to the diagnosis and treatment of related diseases.

## 4 Experimental Section

### Materials and Animals

SIN‐1, ox‐LDL, HPF, and NAC were purchased from Sigma‐Aldrich. Lyso‐Tracker, ER‐Tracker, LDs‐Tracker, and Mito‐Tracker were purchased from Beyotime Biotechnology (Shanghai, China). Liposome synthesis material DSPE‐PEG2000‐NHS was purchased from AVT (Shanghai) Pharmaceutical Tech Co., Ltd. Antibody iNOS and house keeping protein GADPH were brought from Abcam. Human nonsmall cell lung cancer cell line A549 and mouse monocyte macrophage leukemia cell line RAW 264.7 were obtained from the Shanghai Institutes for Biological Sciences (China). BALB/c nude mice (18 ± 2 g) and ApoE genetic defect mice (18 ± 2 g) are bought from Shanghai slaccas experimental animal Co., Ltd (Shanghai, China). All animals are Specific Pathogen Free and performed in accordance with the Guidelines for Care and Use of Laboratory Animals of Xiamen University and the experiments were approved by the Animal Ethics Committee of the Xiamen University (32201145).

### Synthesis of CN‐1

Compound 4‐fluoro‐2‐hydroxybenzaldehyde (100 mg, 0.713 mmol) and K_2_CO_3_ (246.59 mg, 1.78 mmol) were added in 20 mL acetonitrile respectively, stirred for 5 min, and then added compound 4‐(bromomethyl) benzeneboronic acid pinacol ester (211.97 mg, 0.713 mmol), stirred for 24 h. The solvent was then removed under reduced pressure. The product was purified using a silica gel column (dichloromethane (DCM)/MeOH = 50:1) to obtain CN‐1, which was an canary yellow solid (167.2 mg, isolated yield: 65.77%): ^1^H‐NMR (500 MHz, CDCl_3_) *δ* 10.48 (s, 1H), 7.91 (m, 3H), 7.47 (d, *J* = 5.0 Hz, 2H), 6.76 (m, 2H), 5.23 (s, 2H), 1.39 (s, 12H).^13^C‐NMR (125 MHz, CDCl_3_) *δ* 188.18, 168.62, 166.58, 138.31, 135.29, 134.23, 130.88, 126.46, 122.03, 108.46, 100.93, 83.98, 70.81, 24.89. HRMS calculated for C_20_H_22_BFO_4_356.1595, found [M+Na]^+^378.15150 (Figures S33–S35, Supporting Information).

### Synthesis of CN‐2

Compound CN‐1 (100.0 mg, 0.280 mmol) and 0.5 mL Et_3_N were added to the mixed solution of EtOH/DCM (10 mL: 10 mL), stir for 5 min at room temperature, and then benzothiazole‐2‐acetonitrile (48.91 mg, 0.280 mmol) was added to the reaction solution, reacted overnight at room temperature. The solvent was removed under reduced pressure. The product was suspended by 20 mL ethyl acetate (EA), and then filtered to obtain compound CN‐2. Which was an orange solid (128.91 mg, isolated yield: 89.61%):^1^H‐NMR (500 MHz, CDCl_3_) *δ* 8.62 (s, 1H), 8.46 (d, *J* = 10 Hz, 1H), 8.07 (d, *J* = 10 Hz, 1H), 7.90 (t, *J* = 7.5 Hz, 3H), 7.53 (d, *J* = 5 Hz, 2H), 7.49 (d, *J* = 10 Hz, 1H), 7.39 (t, *J* = 7.5 Hz, 1H), 6.59 (d, *J* = 5 Hz, 1H), 6.36 (s, 1H), 5.24 (s, 2H), 1.40 (s, 12H).^13^C‐NMR (125 MHz, CDCl_3_) *δ* 165.57, 160.21155.27, 153.95, 141.20, 139.62, 135.21, 130.32, 126.39, 126.19, 125.08, 123.14, 121.36, 118.18, 111.52, 107.52, 97.32, 83.95, 70.51, 24.91. HRMS calculated for C_29_H_26_BFN_2_O_3_S 512.1741, found [M+H]^+^ 513.18164 (Figures S36–S38, Supporting Information).

### Synthesis of CNP2‐B

Compound CN‐2 (100.0 mg, 0.195 mmol) and 0.5 mL 3,5‐dimethylpiperidine were added to the 10 mL N.N‐dimethylformamide (DMF), reacted overnight at 50 °C. The solvent was extracted by EA and water and then the EA was removed under reduced pressure. The product was purified by a silica gel column (petroleum ether (PE)/EA = 20:1) to obtain compound CNP2‐B. Which was an orange solid (47.28 mg, isolated yield: 40%):^1^H‐NMR (500 MHz, CDCl_3_) *δ* 8.63 (s, 1H), 8.45 (d, *J* = 10 Hz, 1H), 8.07 (d, *J* = 10 Hz, 1H), 7.90 (t, *J* = 7.5 Hz, 3H), 7.53 (d, *J* = 5 Hz, 2H), 7.49 (d, *J* = 5 Hz, 1H), 6.61 (d, *J* = 5 Hz, 1H), 6.38 (s, 1H), 5.33 (s, 2H), 3.77 (d, *J* = 15 Hz, 2H), 2.45 (t, *J* = 12.5 Hz, 2H), 1.73 (s, 2H), 1.49 (m, 2H), 1.39 (s, 12H), 0.97 (m, 6H).^13^C‐NMR (125 MHz, CDCl_3_) *δ* 165.46, 160.27, 160.16, 153.95, 139.66, 135.23, 134.51, 130.37, 126.41, 126.19, 125.11, 123.17, 121.37, 118.13, 107.65, 107.16, 97.71, 83.93, 70.65, 53.43, 42.24, 30.50, 24.90, 19.17. HRMS calculated for C_36_H_40_BN_3_O_3_S 605.2883, found [M+Na]^+^628.28878 (Figures S39–S41, Supporting Information).

### Synthesis of CNP2

Compound 4‐fluoro‐2‐hydroxybenzaldehyde (100.0 mg, 0.714 mmol) and 0.1 mL Et_3_N were added to the mixed solution of EtOH/DCM (10 mL: 10 mL), stirred for 5 min at room temperature, and then benzothiazole‐2‐acetonitrile (124.3 mg, 0.714 mmol) was added to the reaction solution, reacted overnight at room temperature. The solvent was removed under reduced pressure. The product was suspended by 20 mL EA, and then filtered to obtain compound CN‐F. Compound CN‐F (100.0 mg, 0.337 mmol) and 0.5 mL 3,5‐dimethylpiperidine were added to the 10 mL DMF, reacted overnight at 50 °C. The solvent was extracted by EA and water and then the EA was removed under reduced pressure. The product was purified by a silica gel column (PE/EA = 20:1) to obtain compound CNP2‐B. Which was an orange solid (67.85 mg, isolated yield: 51.6%): ^1^H‐NMR (500 MHz, CDCl_3_) *δ* 8.93 (s, 1H), 7.99 (m, 3H), 7.51 (m, 2H), 7.39 (t, *J* = 7.5 Hz, 1H), 6.88 (d, *J* = 10 Hz, 1H), 6.76 (s, 1H), 3.89 (d, *J* = 10 Hz, 2H), 2.51 (t, *J* = 12.5 Hz, 2H), 1.76 (m, 2H), 1.46 (m, 2H), 1.00 (d, *J* = 5 Hz, 6H).^13^C‐NMR (125 MHz, CDCl_3_) *δ* 161.55, 160.90156.89, 154.11, 152.61, 141.77, 136.41, 130.57, 126.13, 124.56, 122.27, 121.62, 111.95, 109.59, 99.38, 54.98, 42.30, 30.65, 19.14. HRMS calculated for C_23_H_23_N_3_OS 389.1562, found [M+H] ^+^390.16333 (Figures S41–S44, Supporting Information).

### Synthesis and Characterization of Probe Gel

50 mg HA‐DA (grafting degree 30%, 446 g mol^−1^, DA content 0.054 mmol) was dissolved in 2 mL pH = 7.4 PBS solution, after fully ultrasonic assisted dissolution with ultrasonic cell breaker, 125 µL 4.05 mol L^−1^ sodium periodate (molar NaIO_4_/DA = 15) PBS solution and 20 µL probe (1 mm) dimethyl sulfoxide (DMSO) solution were added. After shaking and mixing the mixture, the time of hydrogel formation was recorded. After the hydrogel was formed, the injectability and wet adhesion characteristics of the hydrogel were tested. After frozen of the hydrogel, it was put into a lyophilizer for freeze‐drying to remove water while maintaining the 3D structure of the hydrogel. The freeze‐dried hydrogel block was taken out, the cross‐section was cut with a blade, and the cross‐section was observed with SEM and AFM.

### Solvent Sensitivity Experiment

First, 1 mm probe mother liquor was prepared with methanol. 3 mL methanol: water ratio of 99% to 1% solvent system was prepared in a 4 mL doffer tube. 30 µL mother liquor was added into each tube to prepare 10 µm final probe concentration. In addition, DMSO was used to prepare 1 mm probe mother liquor. Toluene, chloroform, dichloromethane, ethyl acetate, acetone, acetonitrile, DMSO, DMF, methanol, and water were added respectively in a 4 mL doffer tube. 30 µL mother liquor was added into each tube to prepare final probe concentration with 10 µm. The UV visible absorption spectrum and fluorescence emission spectrum of the probe under different solvent conditions were measured by UV visible spectrophotometer and fluorescence spectrometer, respectively. The excitation wavelength of the fluorescence spectrum is 430 nm. DFT calculation of the probe molecular structure was carried out using the Gaussian software B3LYP/6‐31G program, the electron cloud density, intramolecular charge transfer, HOMO, and LUMO of the structure were analyzed on the basis of theoretical structure optimization, and the theoretical excitation spectrum and emission spectrum of probe were obtained.

### Preparation of LDs Mimics and Peroxynitrite

Commercialized liposomes were used as LDs mimics with an average particle size of 100 nm. Peroxynitrite preparation: 10 mL 0.6 m NaNO_2_ was taken and 10 mL 0.7 m H_2_O_2_ was added, stirred at 0 °C for 5 min, 10 mL 0.6 m HCl was added quickly while stirring, then 20 mL 1.5 m NaOH was added quickly, stirred continuously for 5 min and mixed well, 500 mg manganese dioxide was added and stirred for 10 min to remove excess H_2_O_2_, the mixed solute was filtered onto removed manganese dioxide. Finally, the peroxynitrite was obtained, and stored at −20 °C for standby after subpackaging. The concentration of ONOO^−^ was determined by measuring the absorption of the solution at 302 nm before use and the extinction coefficient at 302 nm was *ε* = 1670 cm^−1^ m ^−1^.

### Lipid/ONOO^−^ Response Test

Lipid drop simulant selectivity experiment: 1 mm probe mother liquor was prepared with DMSO, PBS was inhaled in a 4 mL doffer tube, preparing 200 µg mL^−1^ LDs mimics respectively: DNA, RNA, BSA, HSA, adenosine triphosphate (ATP), adenosine diphosphate (ADP), glutathione (GSH), Cys, and Glucose. 30 µL probe mother liquor was added into each tube to prepare 10 µm probe final concentration for spectral test. ONOO^−^ selective experiment: PBS was inhaled into a 4 mL doffer tube and 100 µm ClO^−^, H_2_O_2_, IO_4_^−^, NO_2_^−^, Ca^2+^, Cu^2+^, Fe^3+^, Zn^2+^, and K^+^ solution was prepared. 30 µL probe mother liquor was added into each tube to prepare 10 µm probe final concentration for spectral test. Dynamics experiment: 200 µg mL^−1^ LDs mimics and 10 µm ONOO^−^ mixed solution were prepared, 10 µm probe solution was added. Spectral scanning shall be conducted every 10 min for 2 h to obtain time dependent spectral change data. Titration test: First, it was prepared from 0 to 200 µg mL^−1^ LDs mimics solution of different concentrations, with the final concentration of 10 µm probe solution and ONOO^−^ for spectral scanning. Second, ONOO^−^ was prepared with different concentrations (0–10 µm), the final concentration added was 10 µm probe solution and 200 µg mL^−1^ LDs mimics, and spectral scanning was performed.

### Evaluation of Probe imaging Performance in Cells

Imaging performance of the probe in cells was investigated by culture of A549 cells as a tool cell. 1 × 10^4^ A549 cells were inoculated in a glass plate, normal cultivation in 37 °C incubator for 24 h. First, probe CNP2 and CNP2‐B were prepared into 1 mm mother liquor with DMSO, incubated with commercially available lipid probe, mitochondrial probe, endoplasmic reticulum probe, or lysosomal probe for 1 h respectively. Second, A549 cells were preincubated with SIN‐1 for 2 h and incubated with CNP2‐B probe and other organelle probes for 1 h. After that, the culture dish was washed with a small amount of PBS, 4% paraformaldehyde was added for fixation, and the fluorescence was observed in Leica SP8 confocal system.

### Foam Cell Imaging

Mouse monocyte macrophage leukemia cells RAW 264.7 were cultured, 1 × 10^4^ A549 cells were inoculated in a glass plate, normal cultivation in 37 °C incubator for 24 h. ox‐LDL (10 µg mL^−1^) used to incubate macrophages for 24 h to induce foam cell phenotype generation. 2 µg mL^−1^ LPS or 100 nm PMA were used to incubate RAW 264.7 cells for 24 h to induce inflammatory reaction. Western blotting was used to investigate the expression of iNOS in cells. Detection kit was used to detect the content of NO, ONOO^−^, and ROS in cells. 10 µm lipid probe solution was incubated with cells for 1 h, and the number, shape, and size of lipid droplets in cells were observed under confocal microscope. The cells were incubated with 10 µm CNP2‐B and HPF for 1 h respectively, fixed with 4% paraformaldehyde, observed fluorescence in Leica SP8 confocal system.

### Carotid Atherosclerotic Plaque Imaging

ApoE genetically defective C57BL/6 mice were prepared to build carotid atherosclerosis model. After carotid artery ligation and high‐fat diet for one month, a mouse model of carotid atherosclerosis was build. The carotid artery was removed and frozen sections were prepared, oil red O staining was used to analyze the formation of atherosclerotic plaque. After the carotid atherosclerosis model is successfully constructed, the carotid artery was exposed, probe‐loaded hydrogel was applied on the surface of blood vessel, incubated for 10 min, then the probe‐loaded hydrogel was removed by filter paper adsorption, then the fluorescence of the blood vessel position was continuously observed and photographed under the living imaging system for 1 h. Then carotid arteries were removed for imaging. After that, the blood vessels were fixed with 4% paraformaldehyde for 24 h, dehydrated with 40% sucrose for 3 d, and then frozen sectioned. Fluorescence was observed in Leica SP8 confocal system.

### Data Analysis

Data normalization method was used to preprocess data. Results are presented as mean ± S.D. *n* = 6. Error bars represent the S.D. of the mean from independent samples studied in the represented experiments. *P*‐values are calculated using one‐way analysis of variance (ANOVA) with Bonferroni correction, *P < 0.05, **P < 0.01, and ***P < 0.001. Biostatistical analysis software adopts Graph Pad Prism 9.

## Conflict of Interest

The authors declare no conflict of interest.

## Supporting information

Supporting Information

Supporting Information

## Data Availability

Research data are not shared.
