# Supplementary material for: An “AND” Molecular Logic Gate as a Super‐Enhancers for De Novo Designing Activatable Probe and Its Application in Atherosclerosis Imaging
Source: Adv Sci (Weinh). 2023 Feb 19;10(12):2207066. doi: 10.1002/advs.202207066 (PMC10131802; doi:10.1002/advs.202207066)
Supplement: Supplementary file 1 — Supporting Information [file ADVS-10-2207066-s001.pdf]

<Supporting information>

An “AND” Molecular Logic Gate as a Super-enhancers for De Novo Designing Activatable probe and its Application in Atherosclerosis Imaging

Mangmang Sang, Yibo Huang, Lu Wang, Lei Chen, Nawsherwan, Gang Li, Yan Wang, Xiu Yu, Cuilian Dai,* and Jinrong Zheng**

Prof. J. Zheng, Prof. C. Dai, Prof. Y. Wang, Prof. G. Li, Dr. Nawsherwan, Y. Huang, Prof. M. Sang
Institute of Cardiovascular diseases,
Xiamen Cardiovascular Hospital of Xiamen University,
School of Medicine,
Xiamen University,
No. 2999 Jinshan Road, Huli District, XiamenXiamen 361006, China.
E-mail:zhengjinrong@xmheart.com,cuiliand2013@126.com

Prof. X. Yu
Shenzhen Key Laboratory of Respiratory Diseases,
Shenzhen People’s Hospital,
Southern University of Science and Technology,
3046 Shennan East Road, Luohu District, Shenzhen 518055, China;
E-mail:xiuyucpu2015@163.com,

Prof. L. Wang
Nanjing Hospital of Chinese Medicine Affiliated to Nanjing University of Chinese Medicine,
Nanjing University of Chinese Medicine,
No. 157, Daming Road, Qinhuai District, Nanjing 210000, China

Prof. L. Chen
School of Pharmacy,
Gannan Medical University,
No. 1 Medical College Road, Zhanggong District, Ganzhou 341000, China.

Table S1. Pharmacokinetic parameters of CNP2-B. (n=6).

Parameter	Unit	Value
$t_{1/2}$	h	0.224150594
V	(mg/kg)/(μg/mL)	1.095037468
CL	(mg/kg)/(μg/mL)/h	3.386215135
AUC_{0-t}	μg/mL*h	1.476574819
MRT	h	0.32338095
Vss	mg/kg/(μg/mL)	1.095037468

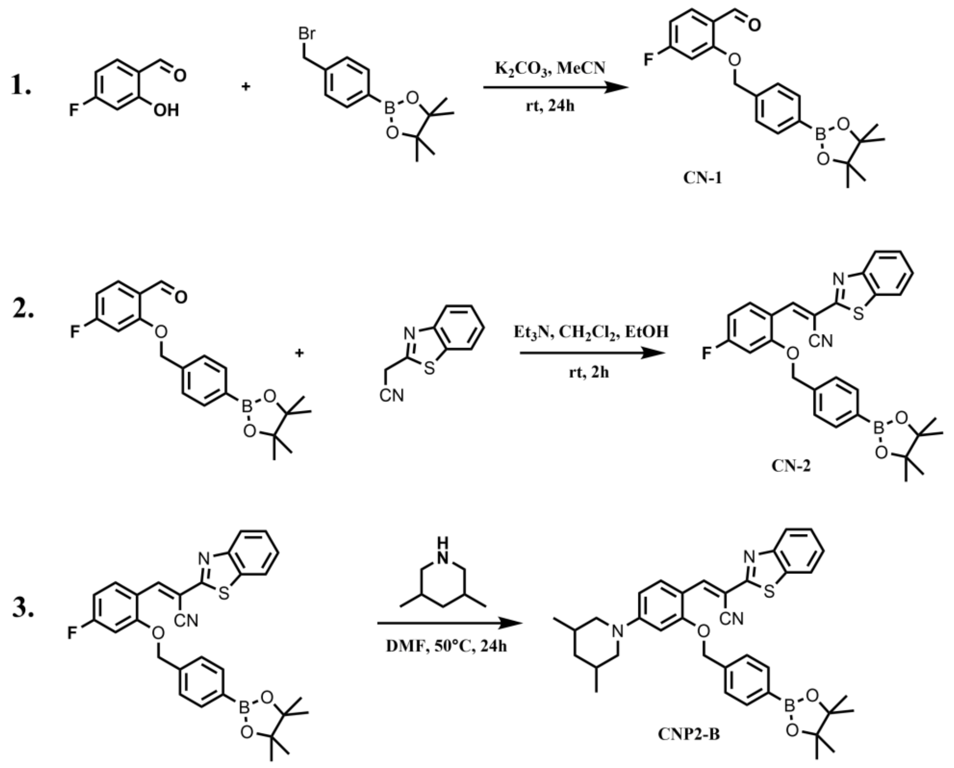

Figure S1. Synthetic route of CNP2-B.

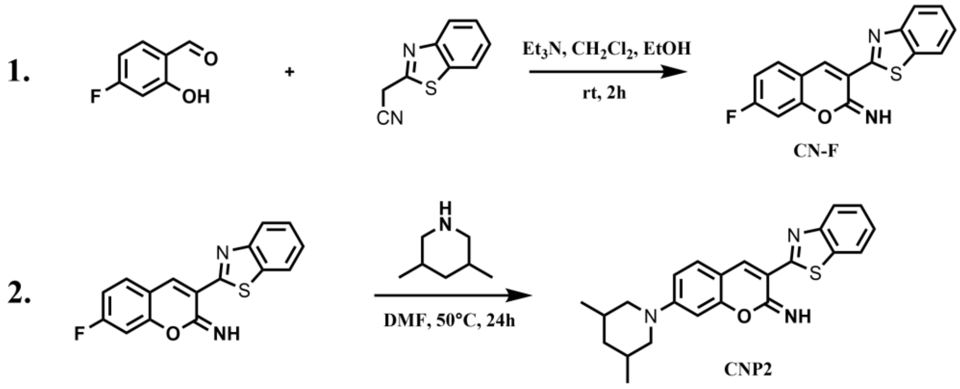

Figure S2. Synthetic route of CNP2.

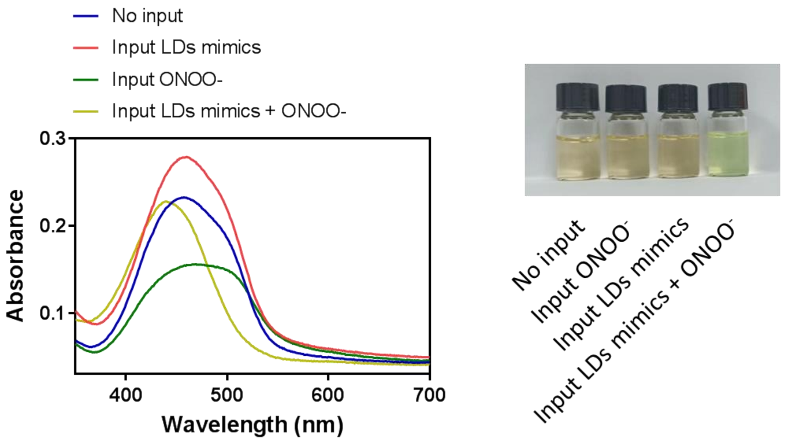

Figure S3. The absorption spectrum and photograph of CNP2-B (10 μM) toward $ONOO^-$ (10 μM) and LDs mimics (200 μg/mL) in PBS, pH = 7.4, DMSO: PBS = 1: 99, 25 ℃, 1h.

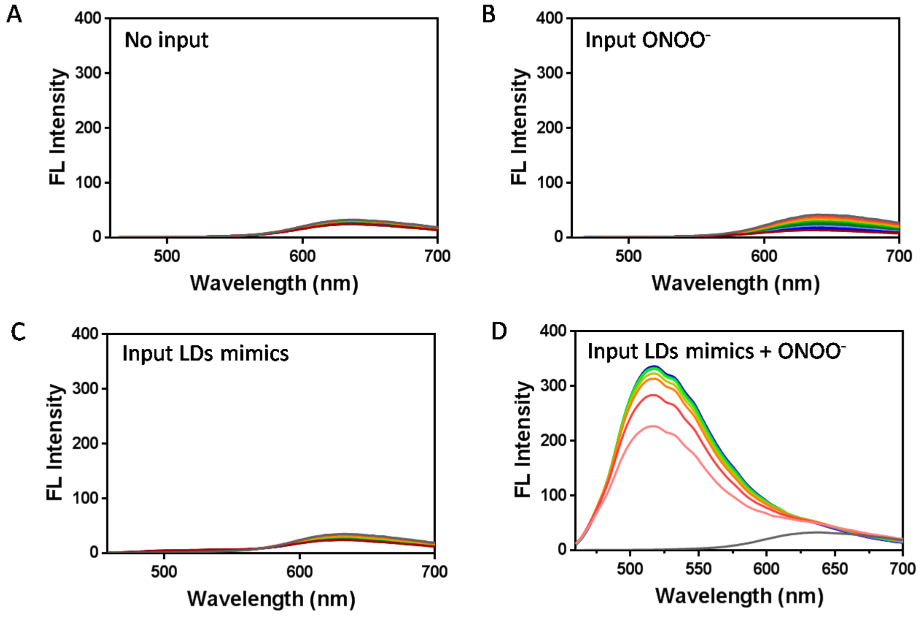

Figure S4. (A-D) The fluorescence spectrum of of CNP2-B (10 μM) toward $ONOO^-$ (10 μM) and LDs mimics (200 μg/mL) in PBS from 0 min to 120 min, pH = 7.4, DMSO: PBS = 1: 99, 25 ℃.

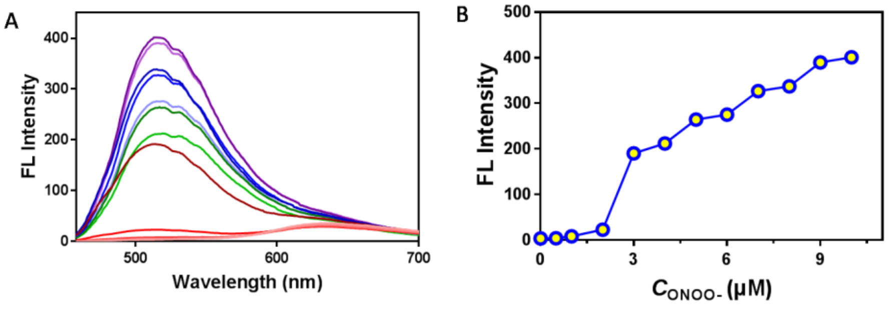

Figure S5. (A) The fluorescence spectrum of CNP2-B (10 μM) toward $ONOO^-$ (0-10 μM) under LDs mimics (200 μg/mL) in PBS, pH = 7.4, DMSO: PBS = 1: 99, 1 h, 25 ℃. (B) The fluorescence intensity of CNP2-B in 517 nm.

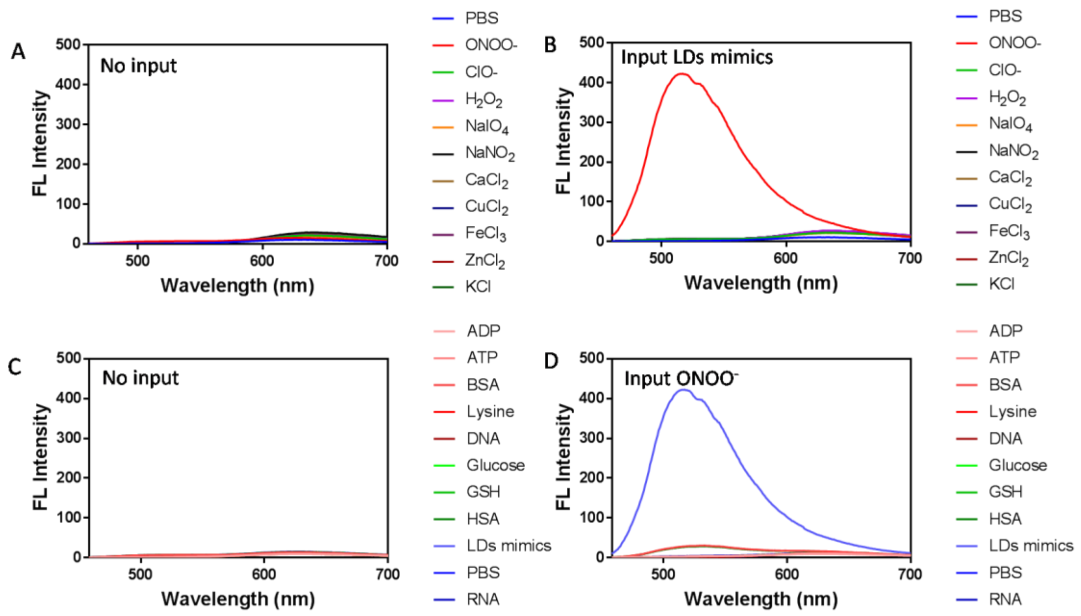

Figure S6. (A) The fluorescence spectrum of CNP2-B (10 μM) toward 10 μM $ONOO^-$, or 100 μM ClO^-, H_2O_2, $NaIO_4$, $NaNO_4$, $CaCl_2$, $CuCl_2$, $FeCl_2$, $ZnCl_2$, KCl in PBS. (B) The fluorescence spectrum of CNP2-B (10 μM) toward 10 μM $ONOO^-$, or 100 μM ClO^-, H_2O_2, $NaIO_4$, $NaNO_4$, $CaCl_2$, $CuCl_2$, $FeCl_2$, $ZnCl_2$, KCl under LDs mimics (200 μg/mL) in PBS. (C) The fluorescence spectrum of CNP2-B (10 μM) toward 200 μg/mL ADP, ATP, BSA, Lysine, DNA, Glucose, GSH, HSA, LDs mimics, RNA in PBS. (D) The fluorescence spectrum of CNP2-B (10 μM) toward 200 μg/mL ADP, ATP, BSA, Lysine, DNA, Glucose, GSH, HSA, LDs mimics, PBS, RNA under $ONOO^-$ (10 μM) in PBS. pH = 7.4, DMSO: PBS = 1: 99, 1 h, 25 ℃.

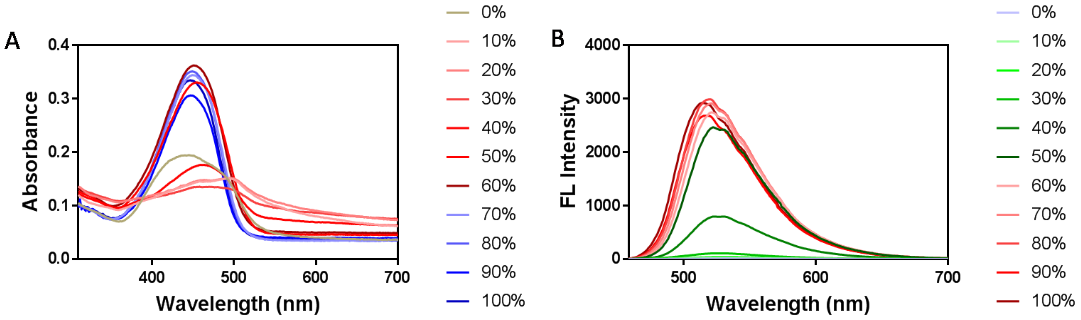

Figure S7. The absorption spectrum (A) and the fluorescence spectrum (B) of CNP2 (10 μM) in MeOH/PBS from 0% to 100%, pH = 7.4, 25 ℃.

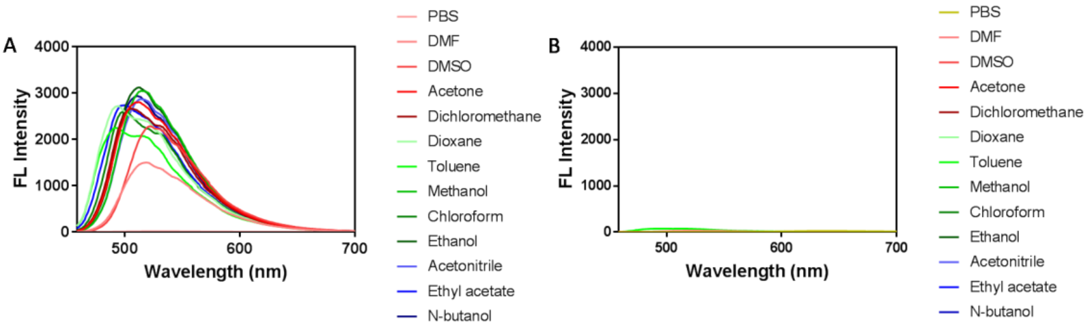

Figure S8. (A) The fluorescence spectrum of CNP2 (10 μM) toward PBS, DMF, DMSO, Acetone, Dichloromethane, Dioxane, Toluene, Methanol, Chloroform, Ethanol, Acetonitrile and Ethyl acetate. (B) The fluorescence spectrum of CNP2-B (10 μM) toward PBS, DMF, DMSO, Acetone, Dichloromethane, Dioxane, Toluene, Methanol, Chloroform, Ethanol, Acetonitrile and Ethyl acetate.

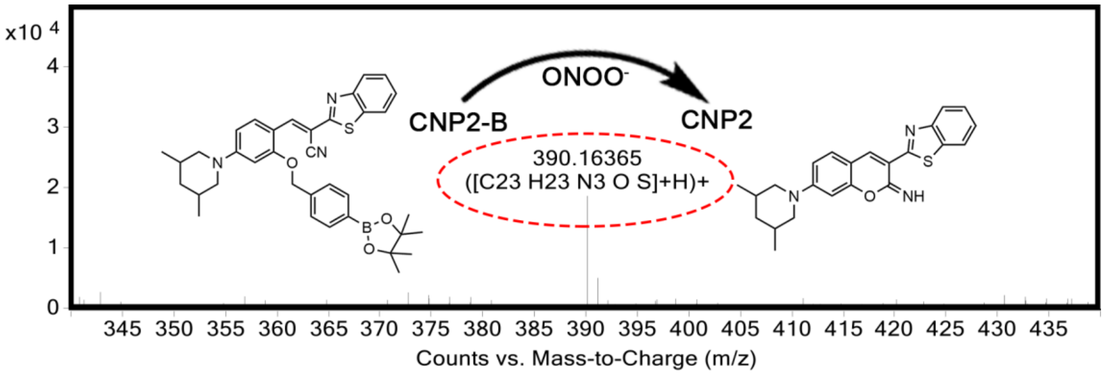

Figure S9. The high resolution mass spectrometry (HRMS) was used to analyse the reaction products of 10 μM CNP2-B toward 10 μM $ONOO^-$ under 200 μg/mL LDs mimics.

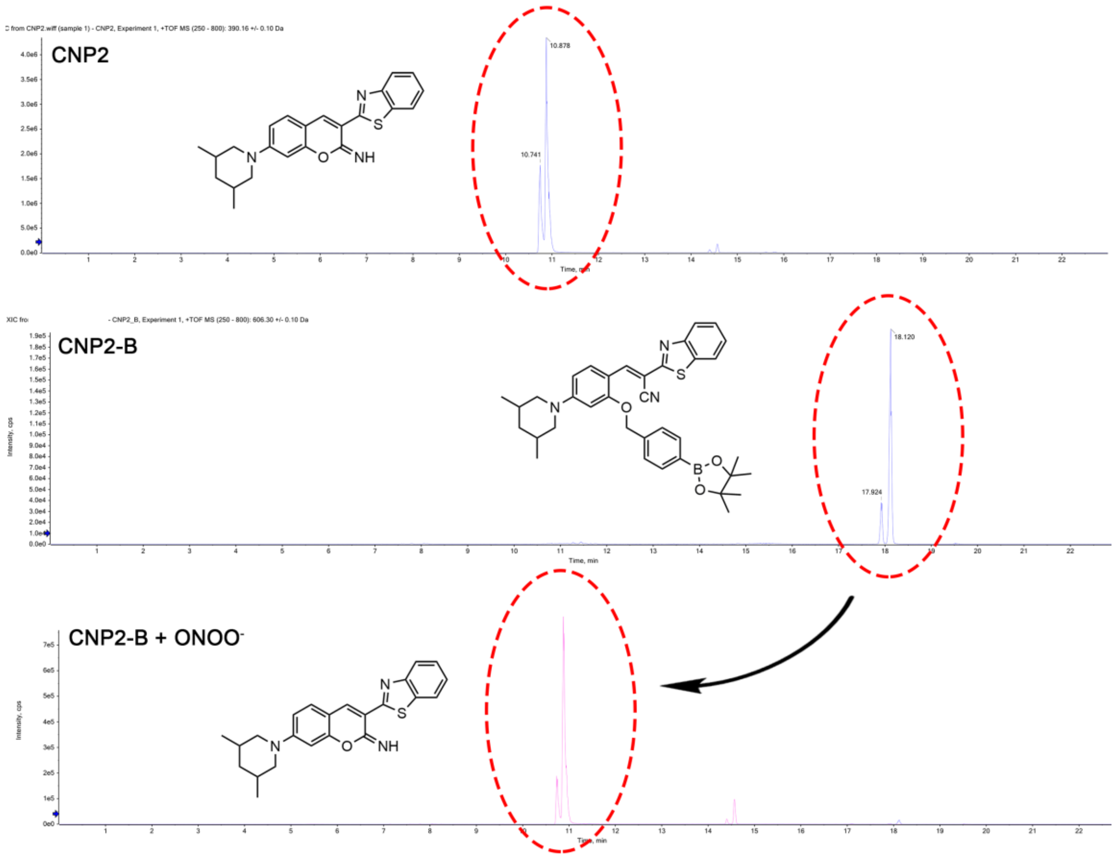

Figure S10. The high performance liquid chromatography (HPLC) was used to analyse the reaction products of 10 μM CNP2-B toward 10 μM $ONOO^-$ under 200 μg/mL LDs mimics.

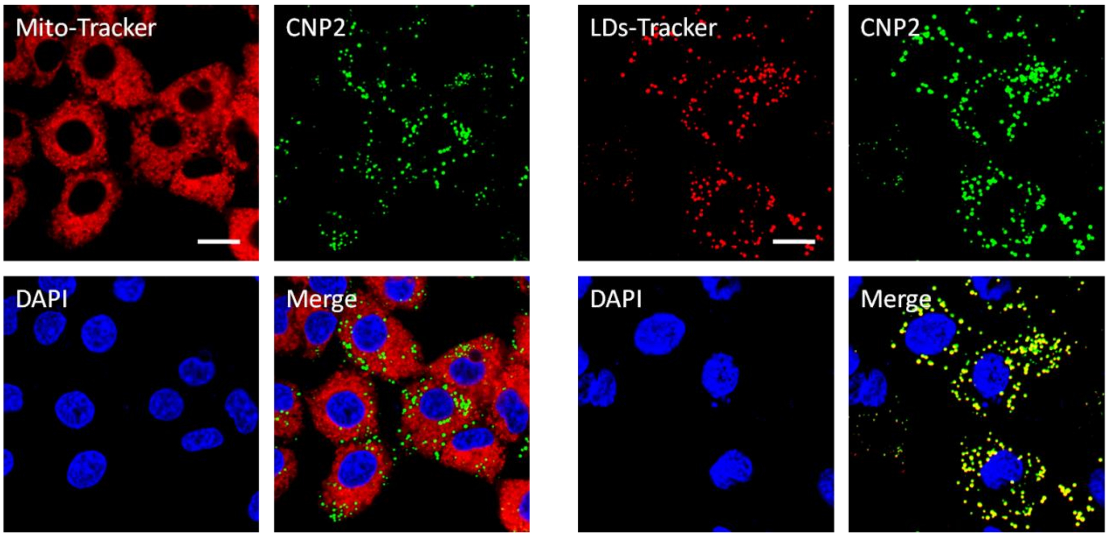

Figure S11. The confocal image of organelles localization of CNP2 in A549. Green channel: λ_{ex} = 488 nm; Red channel: λ_{ex} = 555 nm; Blue channel: λ_{ex} = 405 nm. The bar = 20 μm.

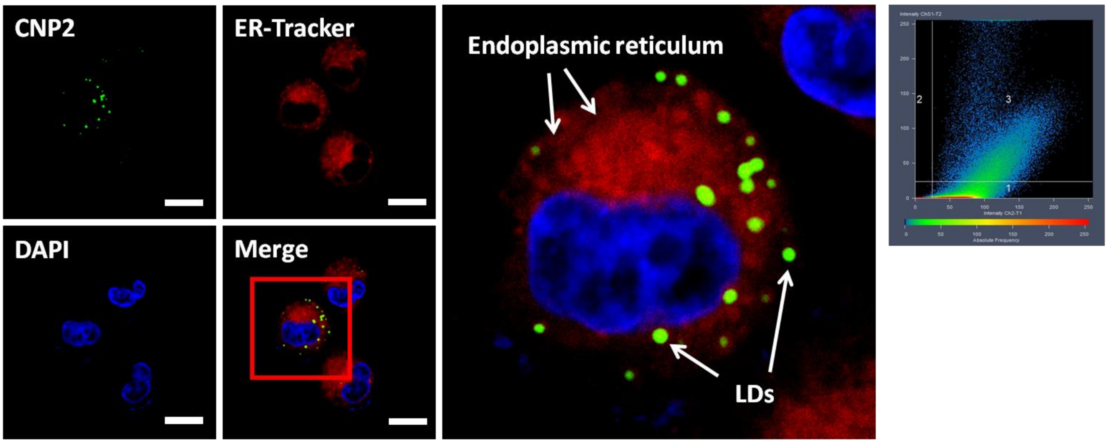

Figure S12. The A549 cells were co-stained with 10 μM CNP2, 10 μM ER-Tracker, and DAPI 1 h, and then the confocal imaging was obtained. Green channel: λ_{ex} = 488 nm; Red channel: λ_{ex} = 555 nm; Blue channel: λ_{ex} = 405 nm. The bar = 20 μm.

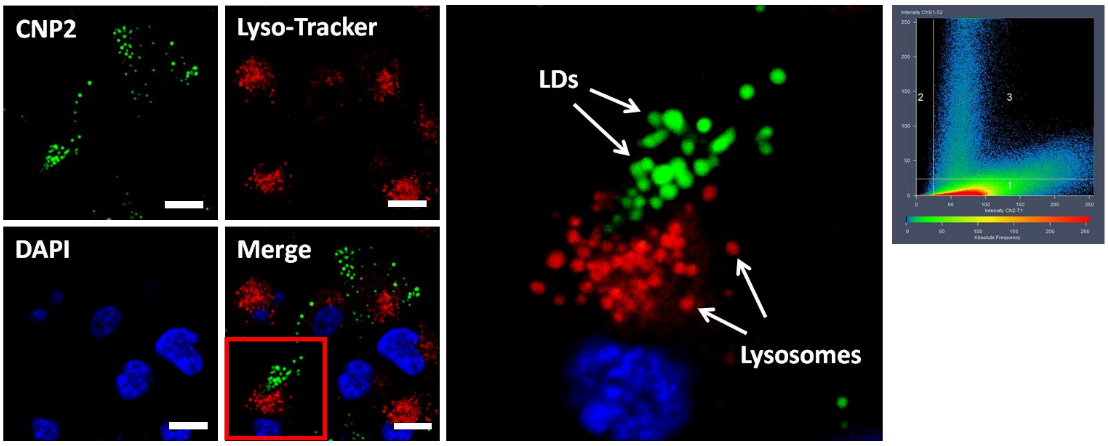

Figure S13. The A549 cells were co-stained with 10 μM CNP2, 10 μM Lyso-Tracker, and DAPI 1 h, and then the confocal imaging was obtained. Green channel: λ_{ex} = 488 nm; Red channel: λ_{ex} = 555 nm; Blue channel: λ_{ex} = 405 nm. The bar = 20 μm.

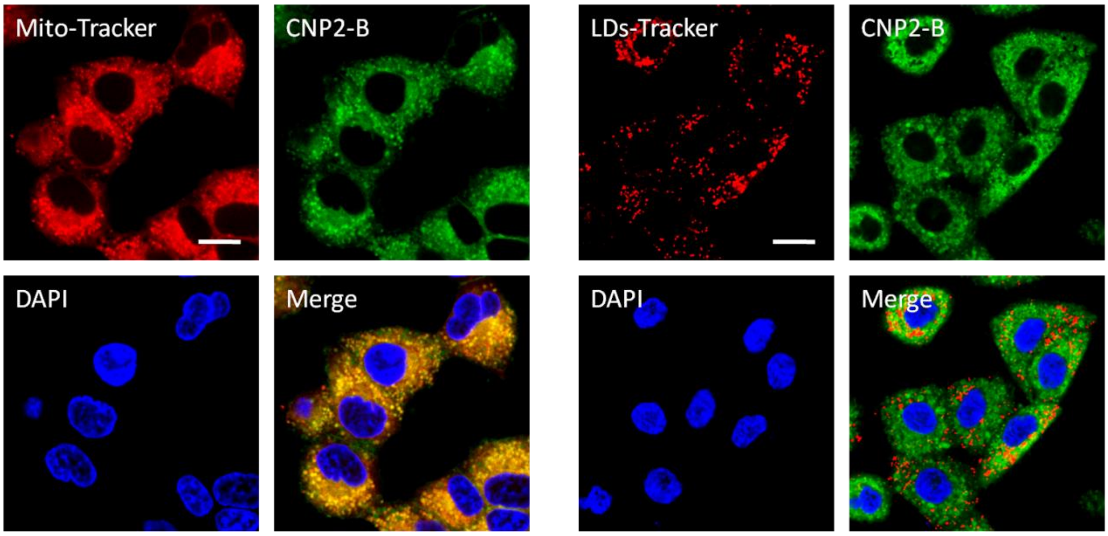

Figure S14. The confocal image of organelles localization of CNP2-B in A549. Green channel: λ_{ex} = 488 nm; Red channel: λ_{ex} = 555 nm; Blue channel: λ_{ex} = 405 nm. The bar = 20 μm.

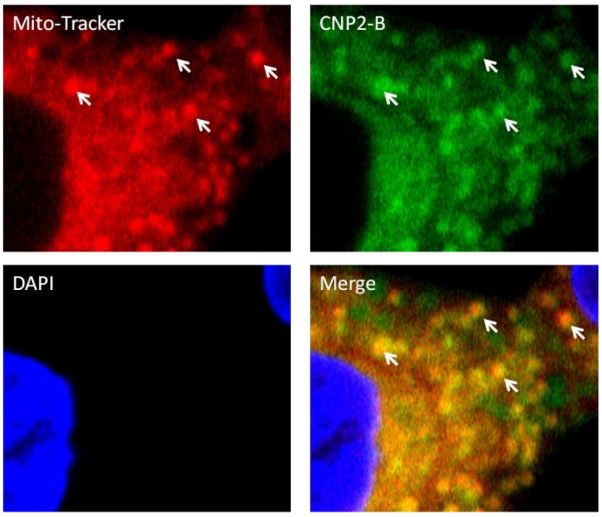

Figure S15. The enlarged image of Figure 3B. Green channel: λ_{ex} = 488 nm; Red channel: λ_{ex} = 555 nm; Blue channel: λ_{ex} = 405 nm.

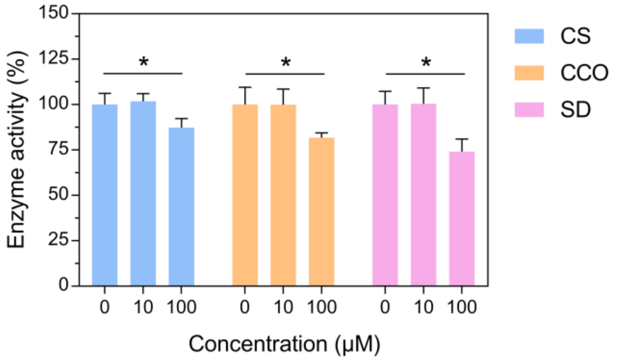

Figure S16. The enzyme activity of citrate synthase (CS), cytochrome C oxidase (CCO), and succinate dehydrogenase (SD) of A549 cells after administration with CNP2-B for 24 h. (n = 6; mean ± SD, *P < 0.05).

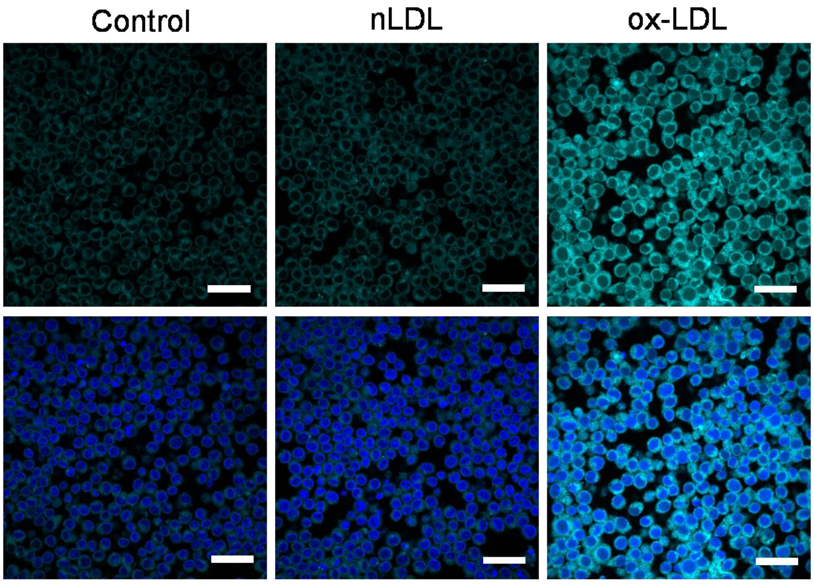

Figure S17. The Raw 264.7 cells were treated with nLDL or ox- LDL 10 μg/mL 24 h, and then incubated with 10 μM DCFH-DA 2 h. The confocal imaging was obtained. λ_{ex} = 488 nm. The bar = 50 μm.

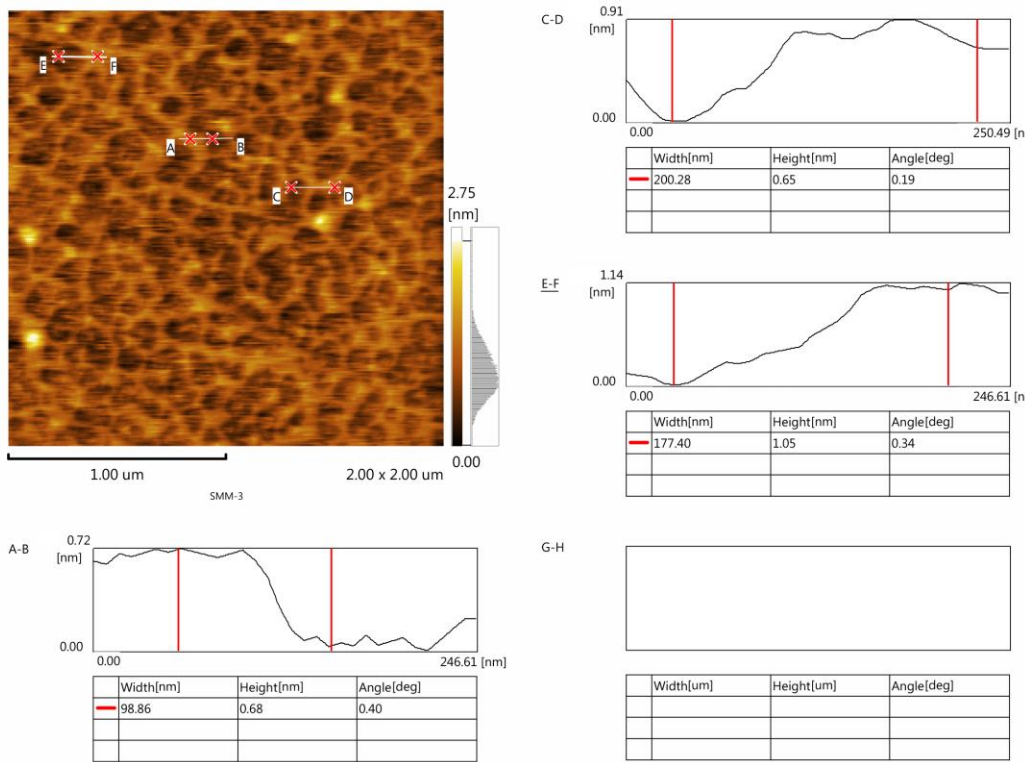

Figure S18. AFM characterization of the CNP2-B loaded hydrogel.

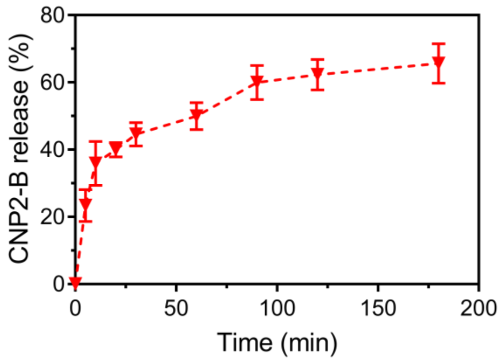

Figure S19. The CNP2-B loaded hydrogel was placed in PBS, and the release efficiency was calculated by measured the absorption intensity of the probe in PBS solution at regular intervals. (n = 6; mean ± SD).

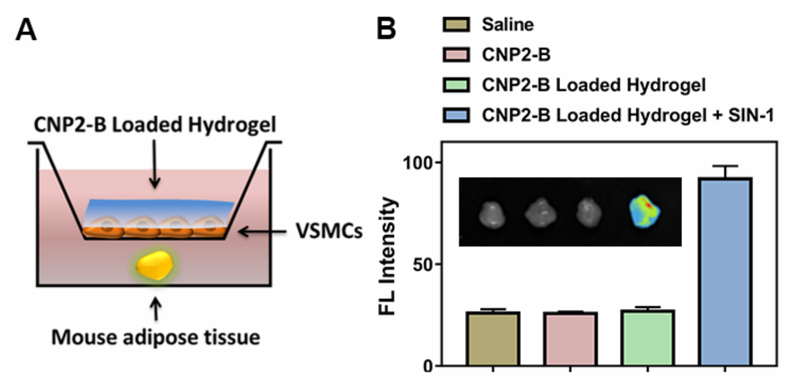

Figure S20. (A) The schematic diagram of transwell assay. (B) The Fluorescence imaging of adipose tissues after transwell assay under different conditions. (n = 6; mean ± SD).

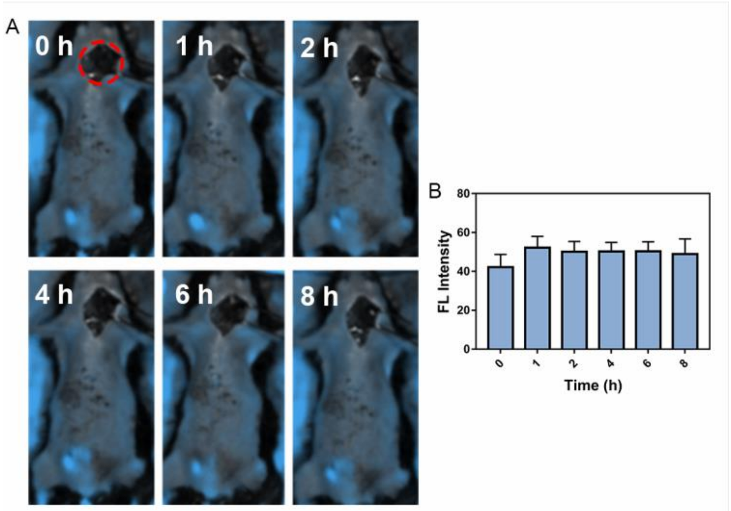

Figure S21. The *in vivo* imaging of carotid atherosclerosis model after injecting 100 μL CNP2-B (10 μM) via tail vein. (n = 6; mean ± SD).

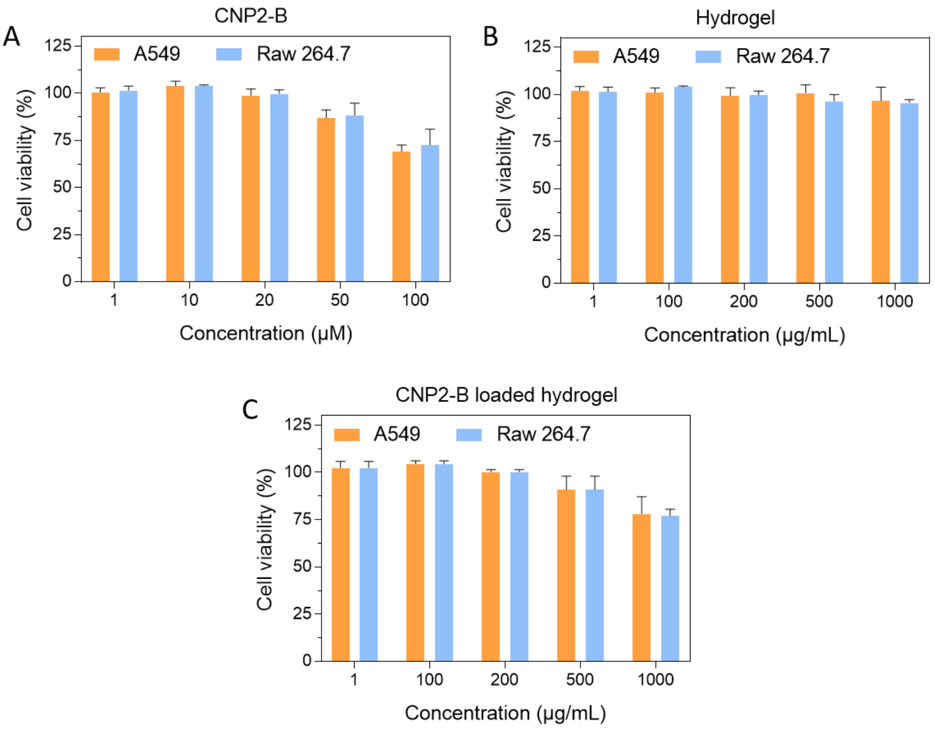

Figure S22. The cell viability of A549 and Raw 264.7 were tested by MTT after administration with CNP2-B (A), Hydrogel (B), or CNP2-B loaded hydrogel. (n = 6; mean ± SD).

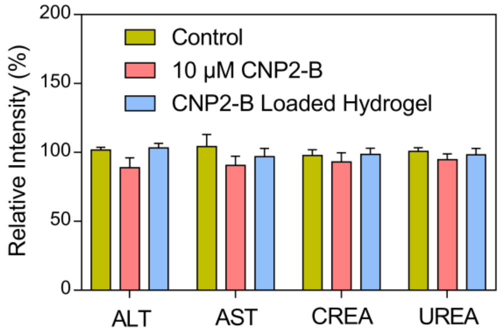

Figure S23. The hepatotoxicity (ALT, AST) and nephrotoxicity (CREA, UREA) were tested after administration with CNP2-B (A), Hydrogel (B), or CNP2-B loaded hydrogel. (n = 6; mean ± SD).

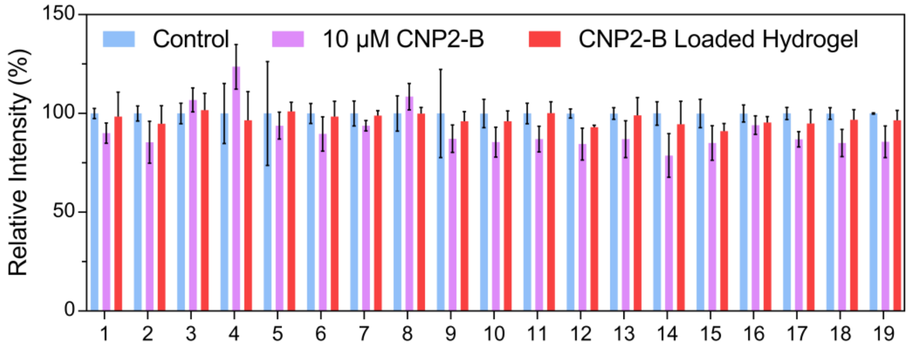

Figure S24. The routine blood tests were conducted after tail vein injection with CNP2-B or *in situ* administration with CNP2-B loaded hydrogel. 1: WBC, 2: NEUT#, 3: LYM#, 4: MONO#, 5: EO#, 6: NEUT%, 7: LYM%, 8: MONO%, 9: EO%, 10: RBC, 11: HGB, 12: HCT, 13: MCV, 14: MCH, 15: MCHC, 16: RDW-CV, 17: PLT, 18: MPV, 19: PDW. (n = 6; mean ± SD).

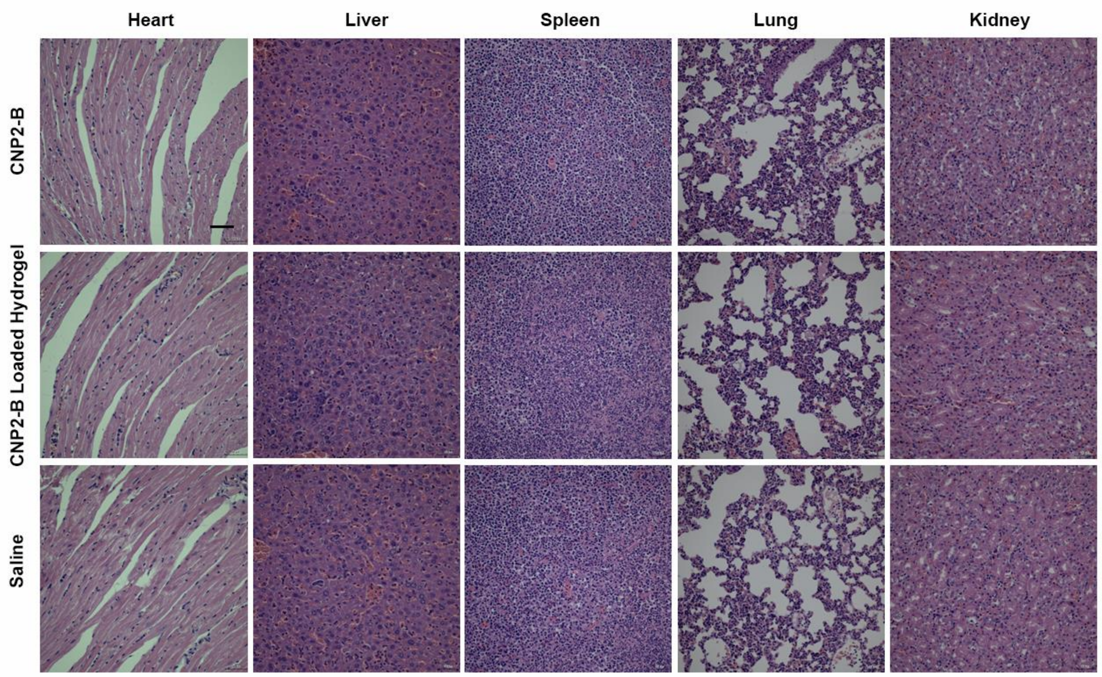

Figure S25. The H&E stain of mouse organs were conducted after tail vein injection with CNP2-B or *in situ* administration with CNP2-B loaded hydrogel.

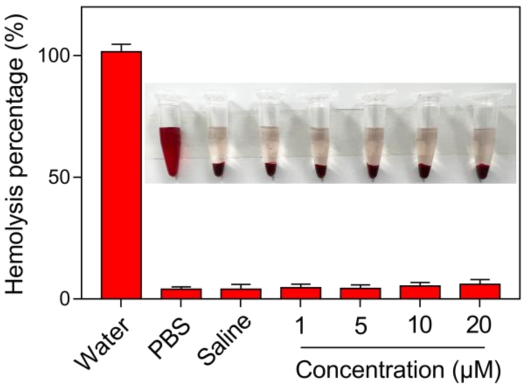

Figure S26. The hemolysis test was conducted under different CNP2-B concentrations. (n = 6; mean ± SD).

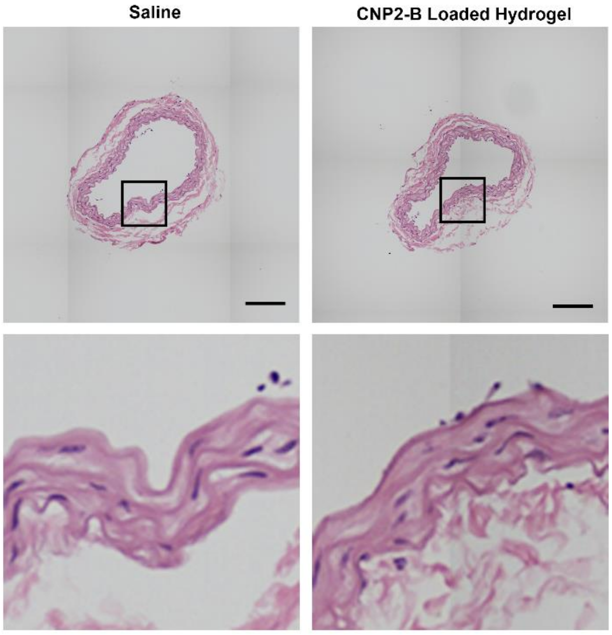

Figure S27. The H&E stain of carotid artery was conducted after *in situ* administration with CNP2-B loaded hydrogel.

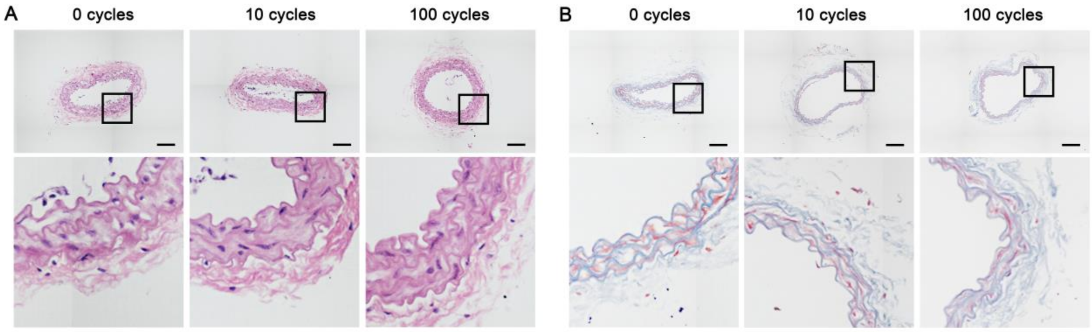

Figure S28. The H&E staining (A) and Masson staining (B) of carotid artery after 460 nm laser irradiation *in vivo*.

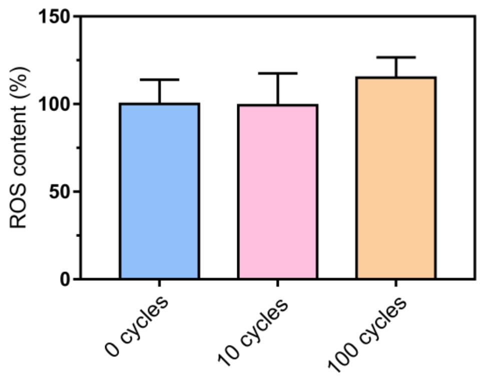

Figure S29. The ROS level of carotid artery was detected after 460 nm laser irradiation *in vivo*. (n = 6; mean ± SD).

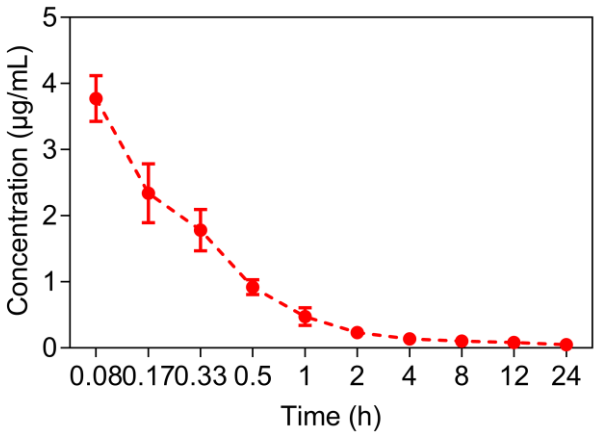

Figure S30. The concentration-time curve of CNP2-B. (n = 6; mean ± SD).

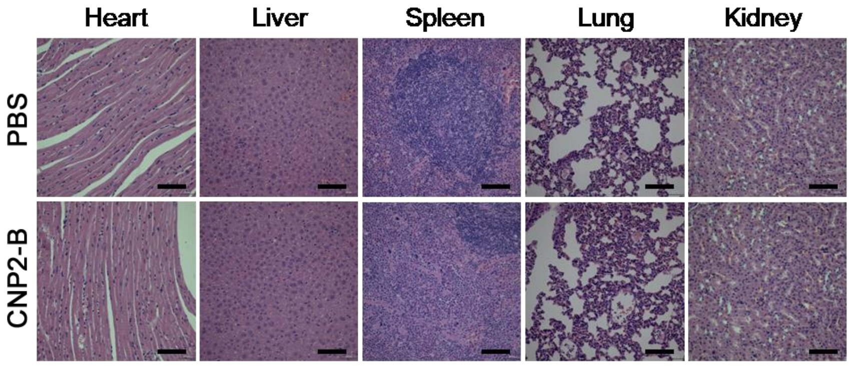

Figure S31. The H-E staining of different organs was evaluated after caudal vein injection of CNP2-B (100 μL, 10 μM) for 5 days.

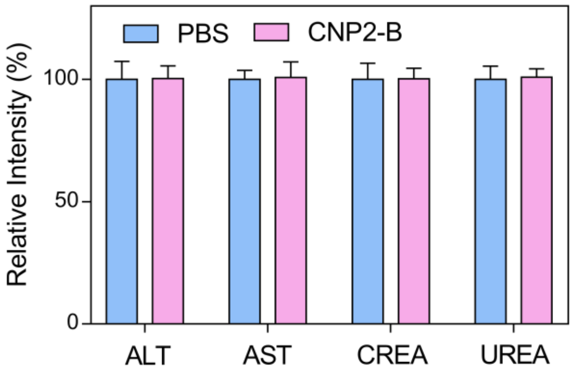

Figure S32. The hepatotoxicity (ALT, AST) and nephrotoxicity (CREA, UREA) of mouse were evaluated after caudal vein injection of CNP2-B (100 µL, 10 µM) for 5 days. (n = 6; mean ± SD).

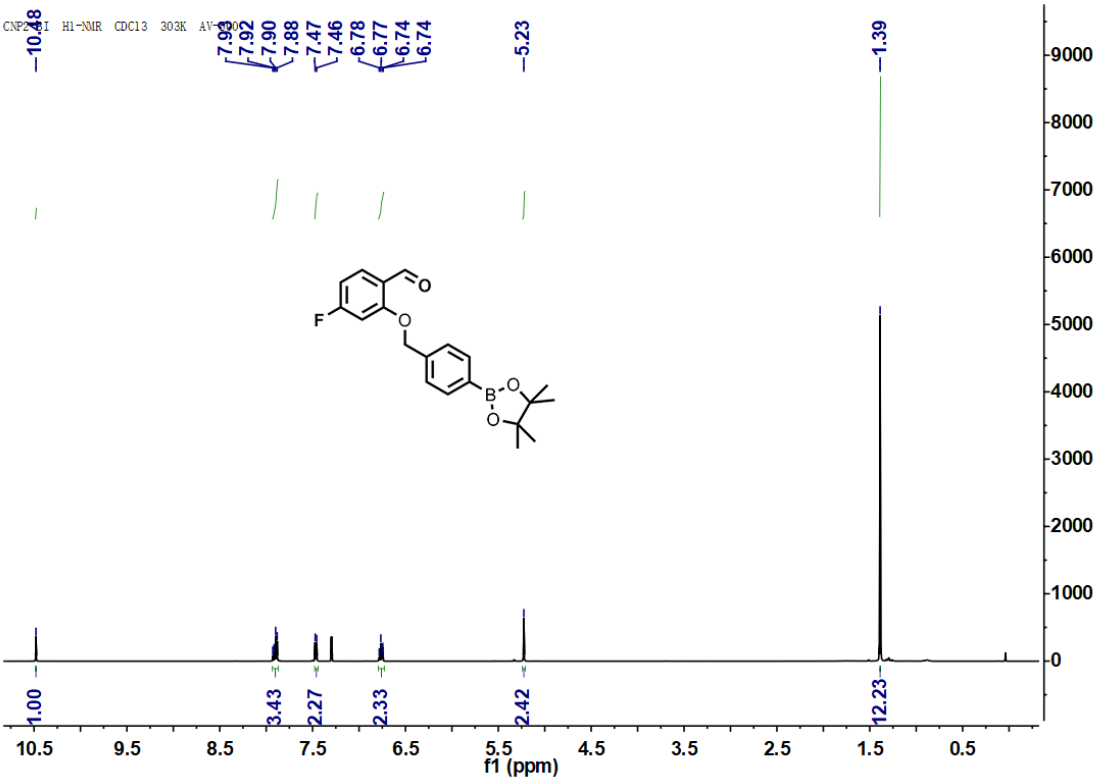

Figure S33. The ^{1}H-NMR of CN-1 in $CDCl_3$.

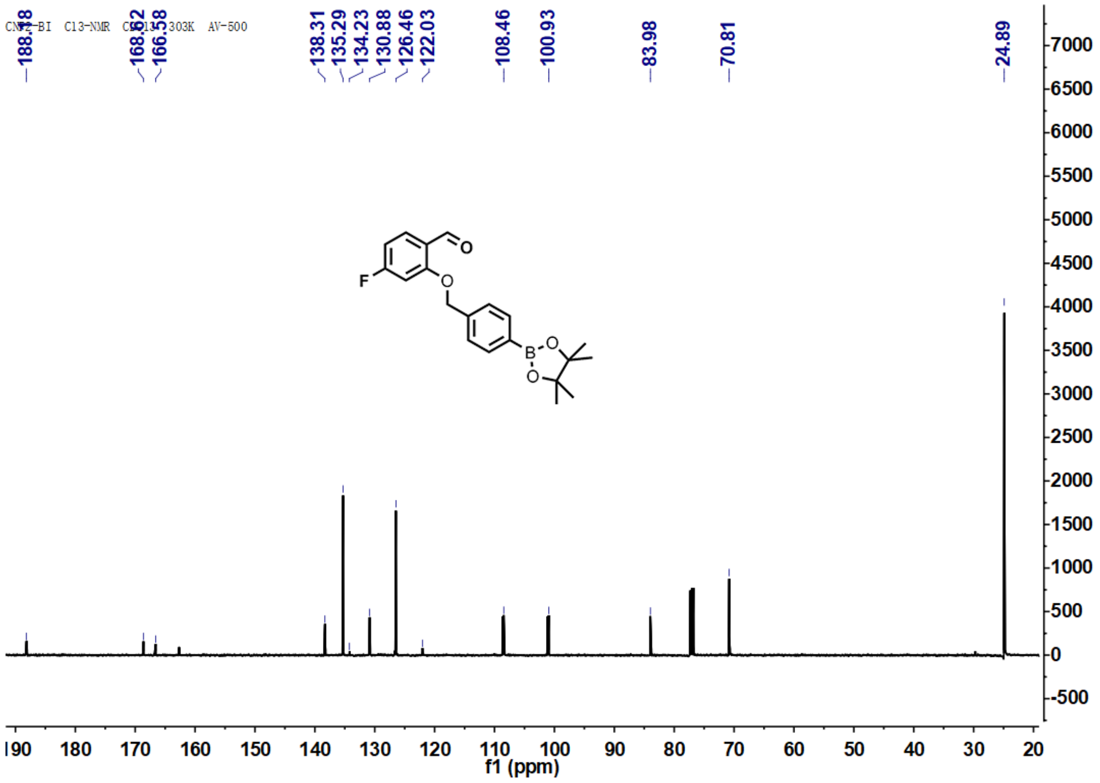

Figure S34. The ^{13}C-NMR of CN-1 in $CDCl_3$.

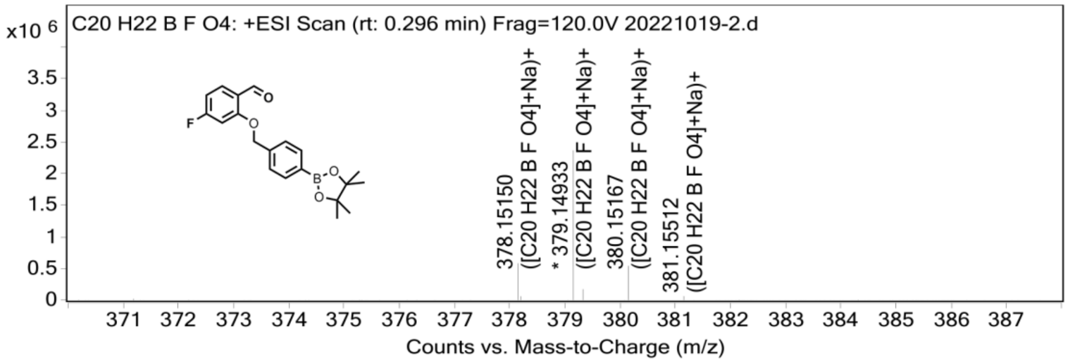

Figure S35. The HRMS of CN-1.

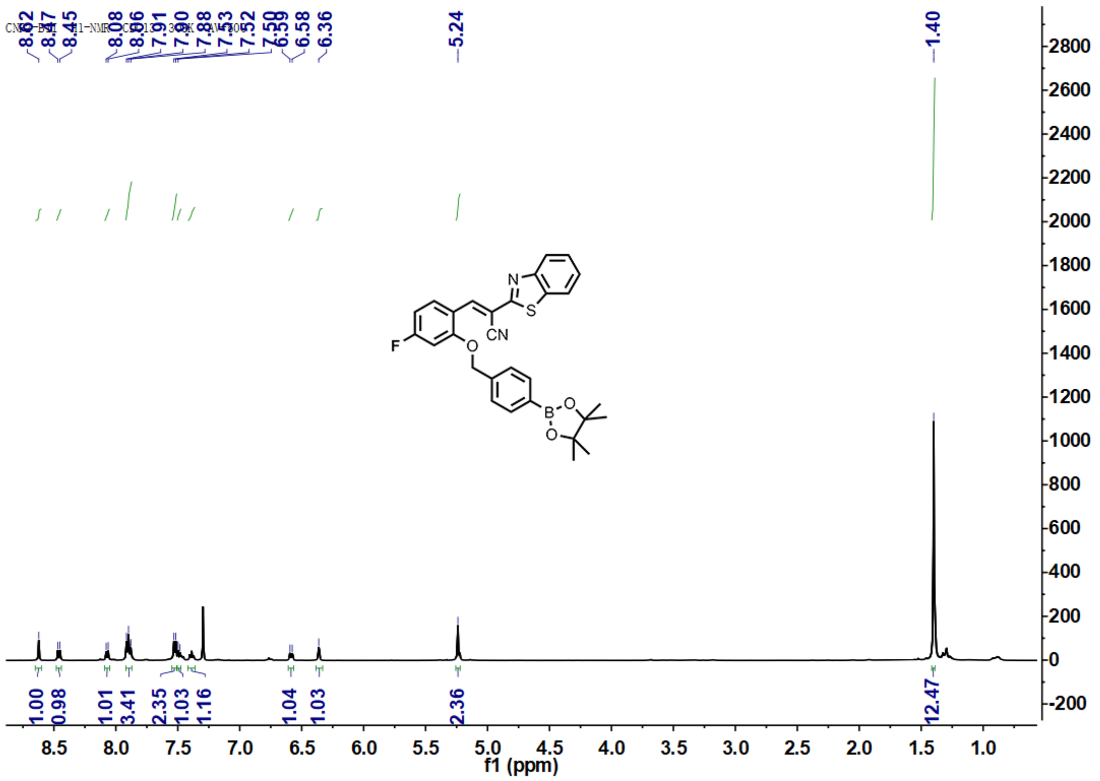

Figure S36. The ^{1}H-NMR of CN-2 in $CDCl_3$.

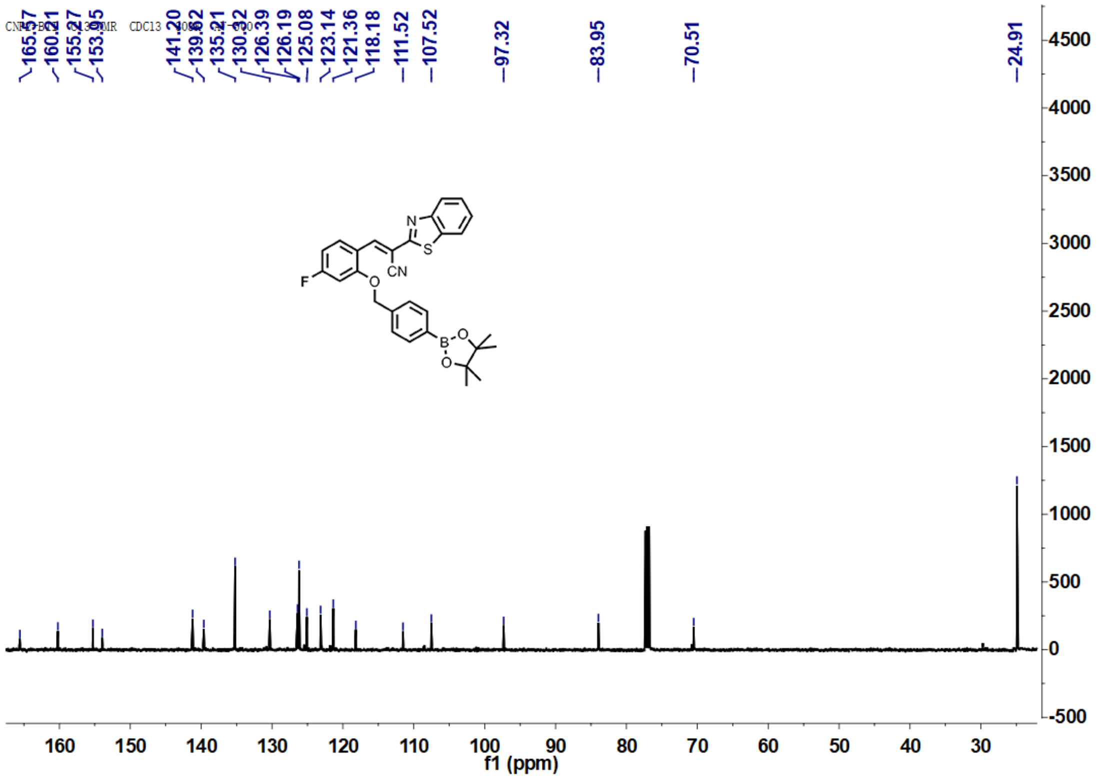

Figure S37. The ^{13}C-NMR of CN-2 in $CDCl_3$.

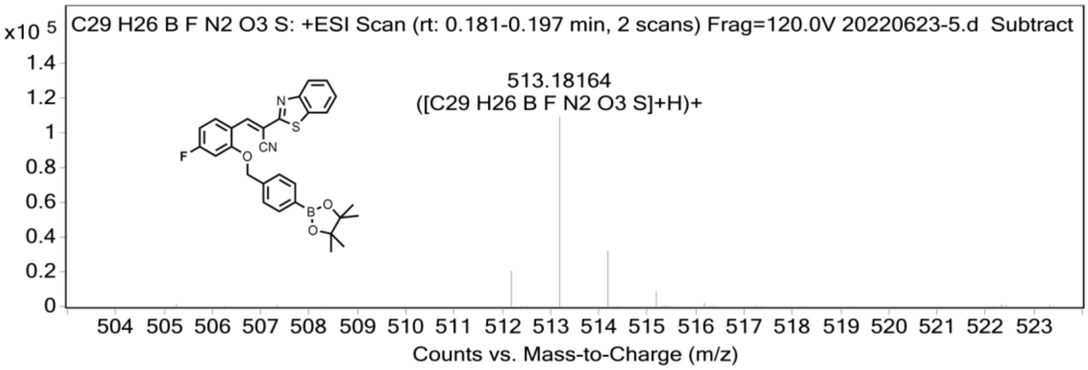

Figure S38. The HRMS of CN-2.

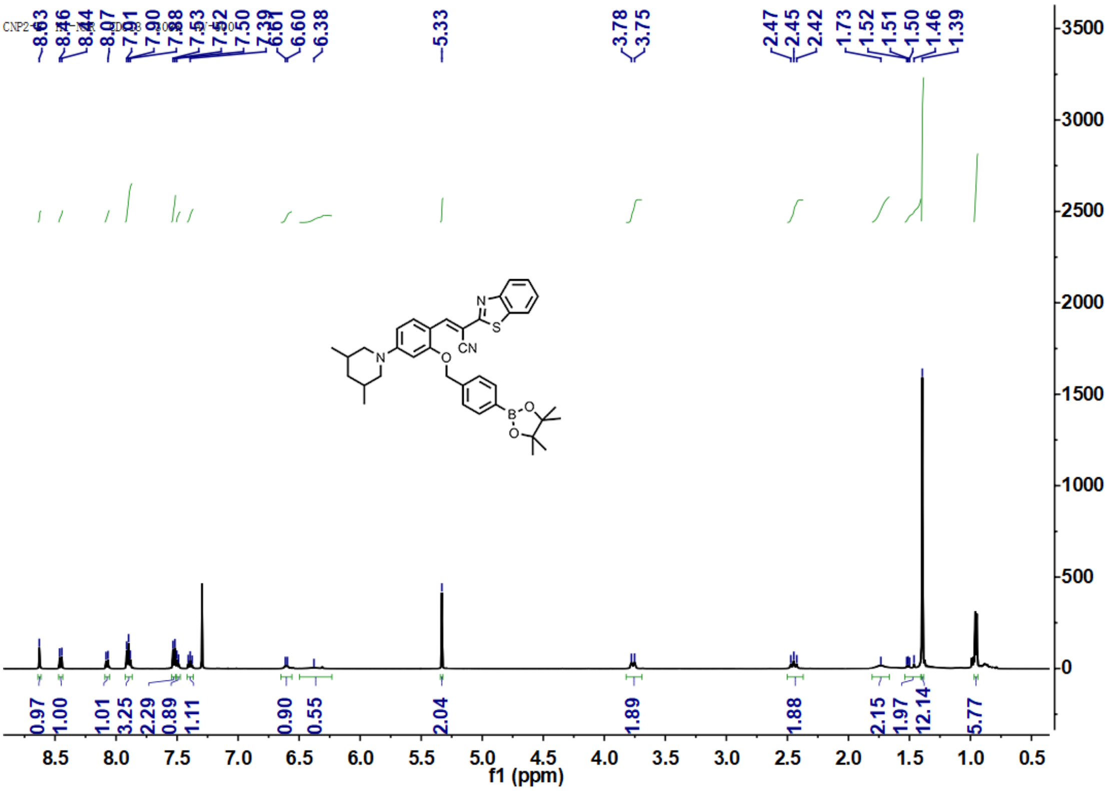

Figure S39. The ^{1}H-NMR of CNP2-B in $CDCl_3$.

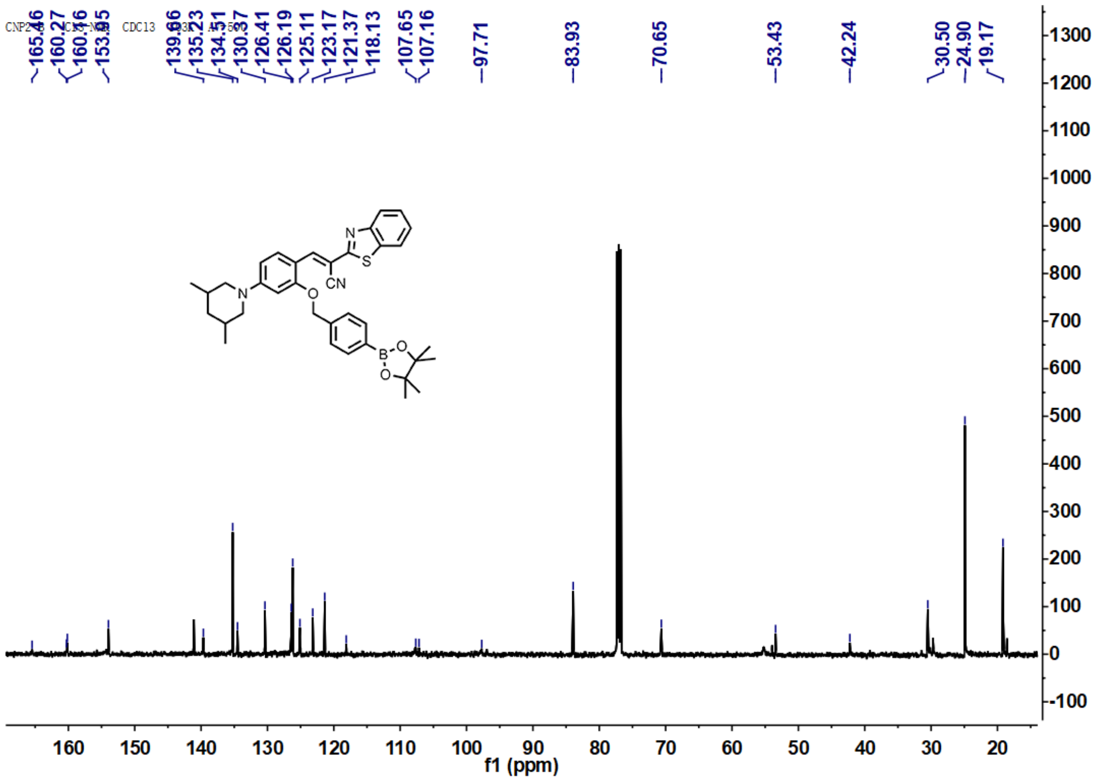

Figure S40. The ^{13}C-NMR of CNP2-B in $CDCl_3$.

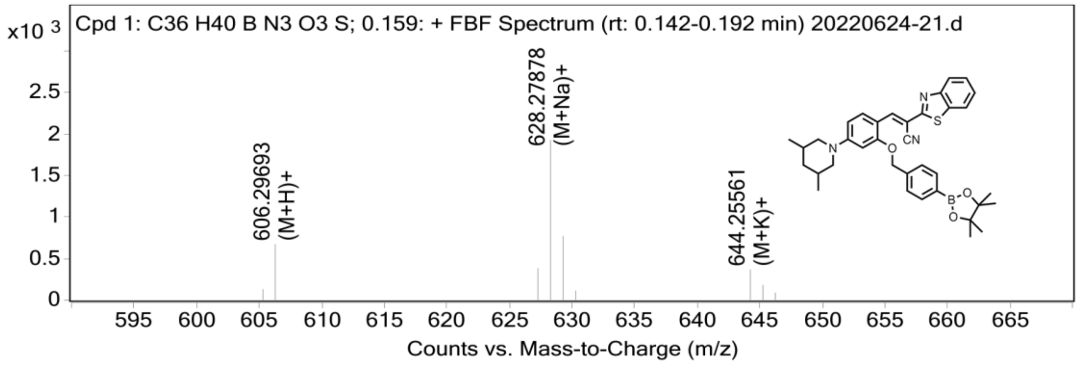

Figure S41. The HRMS of CNP2-B.

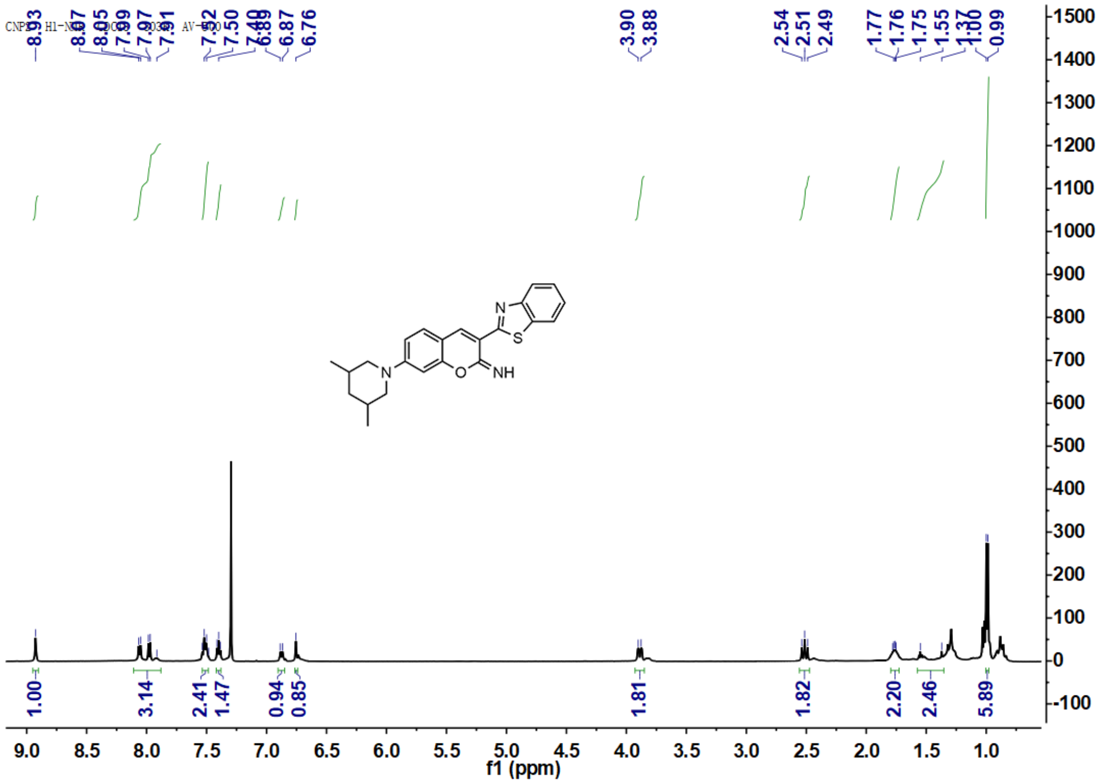

Figure S42. The ^{1}H-NMR of CNP2 in $CDCl_3$.

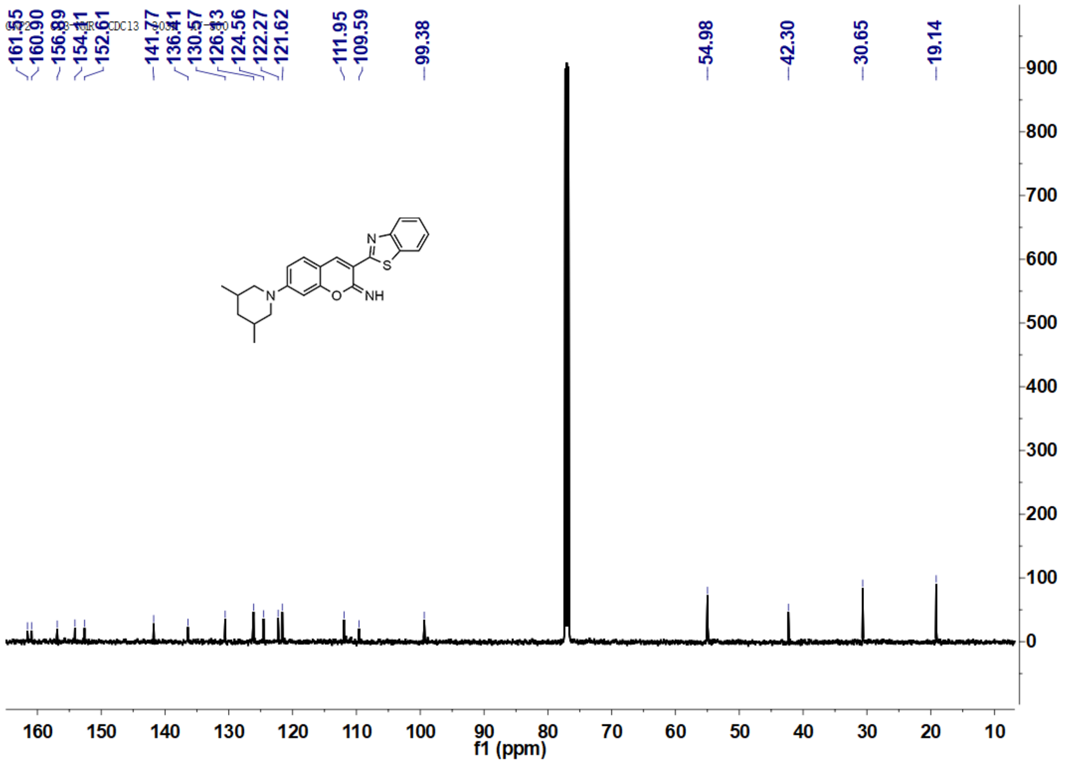

Figure S43. The ^{13}C-NMR of CNP2 in $CDCl_3$.

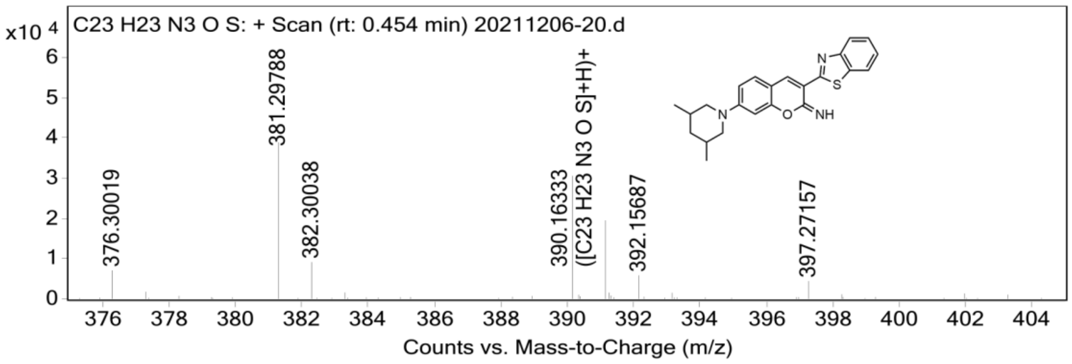

Figure S44. The HRMS of CNP2.
