# Supplementary figures and images for: An “AND” Molecular Logic Gate as a Super‐Enhancers for De Novo Designing Activatable Probe and Its Application in Atherosclerosis Imaging
Source: Adv Sci (Weinh). 2023 Feb 19;10(12):2207066. doi: 10.1002/advs.202207066 (PMC10131802; doi:10.1002/advs.202207066)

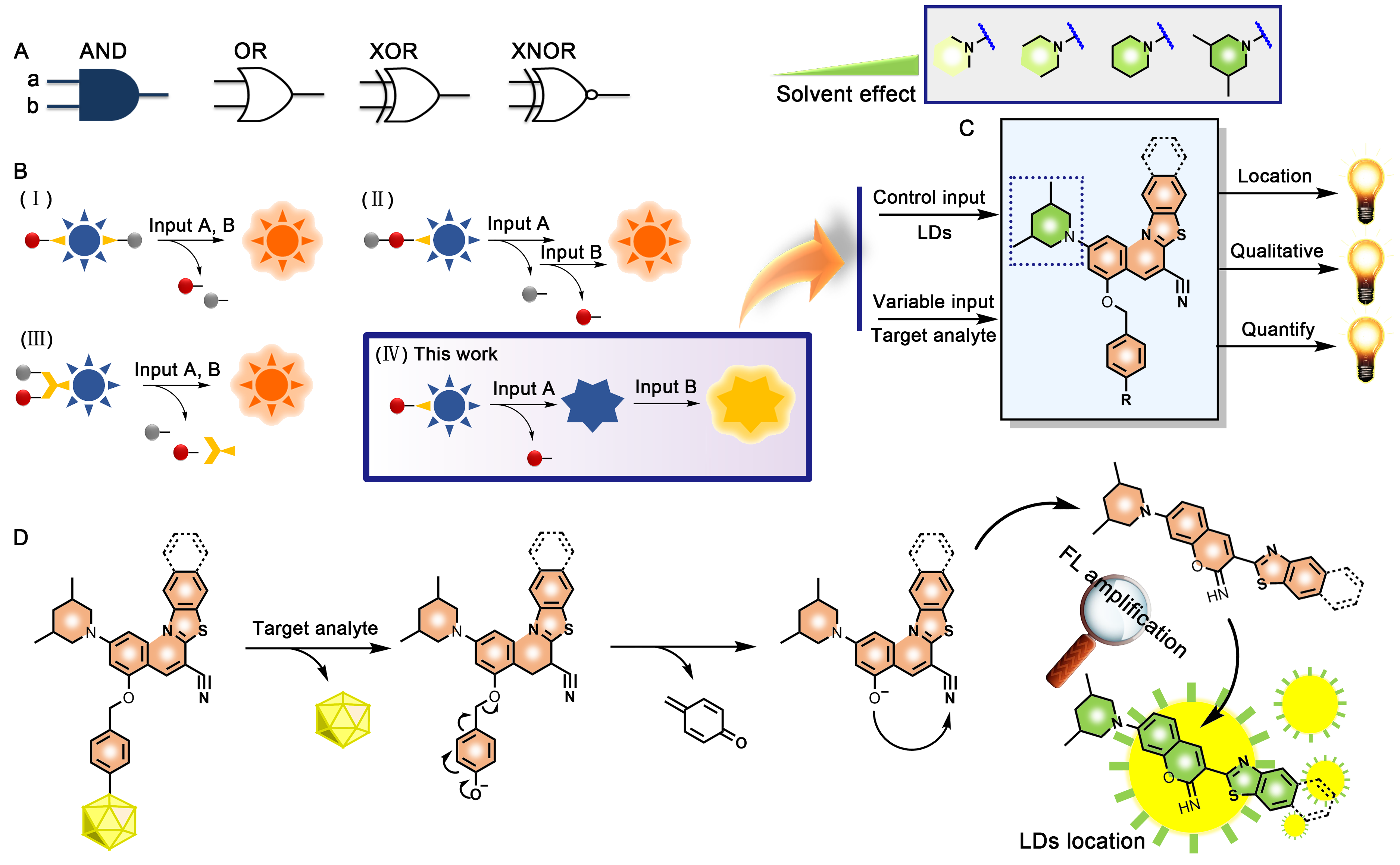

Supplement: Supplementary file 2 — Supporting Information [file ADVS-10-2207066-s002.tif]
